# Single-cell RNA sequencing reveals pro-invasive cancer-associated fibroblasts in hypopharyngeal squamous cell carcinoma

**DOI:** 10.1186/s12964-023-01312-z

**Published:** 2023-10-18

**Authors:** Zhimou Cai, Lin Chen, Siyu Chen, Ruihua Fang, Xiaolin Chen, Wenbin Lei

**Affiliations:** Department of Otolaryngology, The First Affiliated Hospital of Sun Yat-Sen University, No. 58 Zhongshan Er Road, Guangzhou, Guangdong 510080 P.R. China

**Keywords:** Single-cell RNA sequencing, Hypopharyngeal squamous cell carcinoma, Tumor microenvironment, Extracellular matrix cancer-associated fibroblast, Precision therapy

## Abstract

**Background:**

Hypopharyngeal squamous cell carcinoma (HPSCC) has the worst prognosis among all head-and-neck cancers, and treatment options are limited. Tumor microenvironment (TME) analysis can help identify new therapeutic targets and combined treatment strategies.

**Methods:**

Six primary HPSCC tissues and two adjacent normal mucosae from six treatment-naïve patients with HPSCC were analyzed using scRNA-seq. Cell types were curated in detail, ecosystemic landscapes were mapped, and cell–cell interactions were inferred. Key results were validated with The Cancer Genome Atlas and cell biology experiments.

**Results:**

Malignant HPSCC epithelial cells showed significant intratumor heterogeneity. Different subtypes exhibited distinct histological features, biological behaviors, and spatial localization, all affecting treatment selection and prognosis. Extracellular matrix cancer-associated fibroblasts (mCAFs) expressing fibroblast activation protein were the dominant CAFs in HPSCC tumors. mCAFs, constituting an aggressive CAF subset, promoted tumor cell invasion, activated endothelial cells to trigger angiogenesis, and synergized with SPP1^+^ tumor associated macrophages to induce tumor progression, ultimately decreasing the overall survival of patients with HPSCC. Moreover, the LAMP3^+^ dendritic cell subset was identified in HPSCC and formed an immunosuppressive TME by recruiting Tregs and suppressing CD8+ T cell function.

**Conclusions:**

mCAFs, acting as the communication center of the HPSCC TME, enhance the invasion ability of HPSCC cells, mobilizing surrounding cells to construct a tumor-favorable microenvironment. Inhibiting mCAF activation offers a new anti-HPSCC therapeutic strategy.

**Graphical Abstract:**

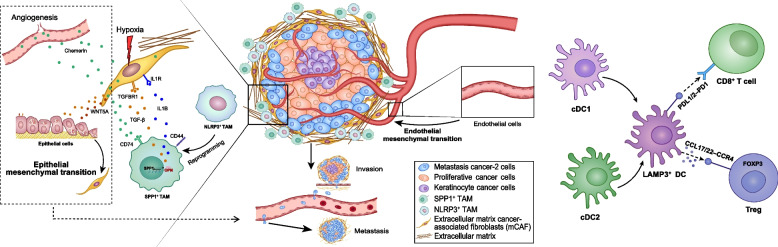

Video Abstract

**Supplementary Information:**

The online version contains supplementary material available at 10.1186/s12964-023-01312-z.

## Background

More than 95% of hypopharyngeal carcinoma cases pertain to hypopharyngeal squamous cell carcinoma (HPSCC), and its annual incidence rate is approximately 0.17–0.8/100,000 (accounting for 1.4–5% of all head-and-neck cancer cases) [1]. As the early symptoms of HPSCC are ambiguous, more than 80% of cases are not diagnosed until the middle or late stages. Furthermore, HPSCC is an aggressive cancer that frequently recurs after therapy, often leading to submucosal dissemination and lymph node metastasis. Currently, HPSCC has the worst prognosis among all head-and-neck cancers [2]; most patients die of HPSCC rather than other causes, with a 5-year overall survival (OS) of only 30–40% [3, 4]. Despite progress in cancer therapy, available treatments for HPSCC have limited efficacy. Therefore, improving patient prognosis is a major challenge [2].

Understanding the tumor microenvironment (TME) is important when aiming to identify effective therapeutic targets. Tumor immunotherapy using immune checkpoint inhibitors has demonstrated the need to target the TME rather than the tumor itself. However, the immunotherapy efficacy for head-and-neck cancer is low [5]. Therefore, in-depth knowledge on tumor heterogeneity in HPSCC, TME, as well as the interaction between HPSCC and their TME is crucial for developing improved HPSCC immunotherapies, benefiting the discovery of novel targets or combined treatment strategies. However, accurately assessing tumor heterogeneity is challenging owing to the limitations of biotechnology techniques [6]. Single-cell RNA sequencing (scRNA-seq) allows researchers to unbiasedly assess heterogeneous cancer and stromal cells at a cellular resolution and investigate molecular components of the TME [7]. However, few studies have investigated HPSCC TME at the single-cell level, as previous scRNA-seq studies of this cancer mainly focused on epithelial cells (EpCs) [8–10]. Thus, the complexity of HPSCC TME and the heterogeneity of its stromal and immune cell subsets remain unclear [11].

Here, we comprehensively investigated HPSCC tumor heterogeneity and TME characteristics via analyzing single-cell transcriptional profiles of 67,025 cells from six HPSCC tissues and two tumor-adjacent tissues. We highlighted the role of extracellular matrix cancer-associated fibroblasts (mCAFs) in HPSCC and identified potential therapeutic targets. Moreover, we generated a potential cell population interaction network centered on mCAFs in the HPSCC TME. These findings will help elucidate HPSCC heterogeneity, with implications for prognosis, diagnosis, and individualized treatment.

## Methods

### Human specimens and ethical approval

All samples were obtained from patients at the First Affiliated Hospital of Sun Yat-sen University (Guangzhou, Guangdong, China). The six enrolled patients were diagnosed with HPSCC; six primary HPSCC tissues along with two adjacent normal mucosa tissues were analyzed. No patient had received antitumor therapy prior to tumor resection. Patient clinical characteristics are summarized in Table S1. All patients provided informed consent. All experimental procedures were approved by the Ethics Board of the IEC for Clinical Research and Animal Trials of the First Affiliated Hospital of Sun Yat-sen University (approval no. [2020]220-1).

### Preparation of single-cell suspensions

Tumor and adjacent tissues were cut into pieces (< 1 mm^3^) in DMEM (Gibco, Germany) with 10% FBS (Gibco) and digested using a MACS tumor dissociation kit (Miltenyi Biotec, Germany) for 30 min on a rotor at 37 °C. The digested mixture was filtered using a 70 µm cell strainer (BD Falcon, USA) in DMEM to obtain dissociated cells. Dissociated cells were centrifuged at 330 × *g* at 4 °C for 10 min. After removing the supernatant, cells were treated with red blood cell lysis buffer (Solarbio, China) for 15 min on ice. After washing twice with PBS, cells were resuspended in sorting buffer (PBS supplemented with 2% FBS). Finally, cells in 10 μL suspension were counted using an inverted microscope and a hemocytometer. Cell viability was assessed via 0.1% trypan blue staining.

### Single-cell RNA sequencing and read processing

Single cells were run on a 10 × Genomics Chromium Controller Instrument, and barcoded scRNA-seq libraries were prepared following the recommended protocol from the Chromium Single Cell 3′ Reagent v3 kit (10 × Genomics) to generate single-cell gel-beads in emulsions (GEMs). Within GEMs, barcoded cDNA was produced via reverse transcription, and GEMs were then broken. The remaining cDNA was fragmented, end-repaired, A-tailed, ligated with adaptors, and amplified via PCR. Finally, every library was sequenced using a NovaSeq 6000 sequencing platform (Illumina, USA), and 150 bp paired-end reads were generated. Raw data were processed using CellRanger (version 5.0.1) to generate gene-barcode matrices (Table S2).

### Quality control and batch effect correction of scRNA-seq data

Gene-barcode matrices were converted into a Seurat object using the “Seurat” R package (version 3.2.2) [12]. Cells with mitochondrial gene percentage > 25% and < 500 detected genes were considered low-quality and removed. To eliminate potential doublets, cells with > 6,000 detected genes were removed, followed by a run through the DoubletFinder package using the default settings [13]. The cell cycle phase prediction score was calculated using Seurat function CellCycleScoring. Next, SCTransform was applied to normalize, scale, and identify variable features of the data and regress out the effects of the cell cycle score (G2M.Score,S.Score), the percentage fraction of mitochondria, the number of features per cell, and the number of unique molecular identifiers per cell [14]. To correct for batch effects, the top 3,000 highly variable genes were used as input in the RunFastMNN function of the SeuratWrappers package [15]. Finally, scaled and batch-effect-corrected expression profiles of all samples were obtained for downstream analyses.

### Dimensionality reduction and clustering

Thirty principal components were selected at a resolution of 0.8, and the FindNeighbors and FindClusters functions in the Seurat package were used for cell clustering. Cells were visualized using UMAP embedding. Previously described marker genes were used to categorize cells into known biological cell types [16]. First, cells were clustered into 11 major cell types. Subsequently, these cell types were divided into subsets for normalization, dimensionality reduction, and further clustering into subclusters, thus allowing for detection of heterogeneity within each cell type. The Seurat Findallmarker function was used to identify differentially expressed genes (DEGs) in each subset or subcluster. The criteria for DEGs were adjusted *P*-value < 0.01 (Wilcoxon rank-sum test) and log_2_ fold-change > 1.

### Copy number variants and differentiation status analysis for cancer cells

The InferCNV R package [17] with default parameters was used to detect initial copy number variants (CNVs) per region in EpCs and to recognize real cancer cells. Immune and stromal cells were used as references.

Transcriptional heterogeneity, developmental potential, and differential status/stemness levels of epithelial subclusters were evaluated using the CytoTRACE R package [18]. Cells were assigned a CytoTRACE score according to differentiation potential, with a higher score indicating higher stemness/fewer differential characteristics.

### Trajectory and RNA velocity analysis

Pseudotime analysis was performed using the Monocle R package [19] to determine the potential lineage differentiation trajectory. Genes with expression that changed along pseudotime were identified using the DifferentialGeneTest function and the formula “ ~ sm.ns (Pseudotime)”. RNA velocity [20] was determined using the velocyto R package to derive the developmental relationships of different cell types, then visualized on a UMAP plot using Seurat.

### Transcription factor regulon analysis

Transcription factor (TF) analysis was conducted as described [21]. We used pySCENIC (version 0.10.2), including the RcisTarget, GRNboost, and AUCell functions, to search against the hg38_refseq-r80_500 bp_up_and_100 bp_down_tss databases for predicting TF activity. The input matrix was a normalized expression matrix from Seurat.

### Pathway analysis

Gene set variation analysis (GSVA) was performed using the GSVA R package [22] to estimate pathway activity of cell groups. Gene sets of curated signaling pathways were downloaded from the Molecular Signatures Database (MSigDB, https://www.gsea-msigdb.org). Pathways with significantly different activity scores were selected using the limma R package.

### Signature scoring of cell subsets

Signature scores were determined by calculating the means of scaled and centered expression values across multiple signature genes using the “AddModuleScore” function in Seurat. The signature gene list for TMA M1 polarization, M2 polarization, angiogenesis, and phagocytosis scores as well as Dendritic cell (DC) activation, migration, and tolerogenic scores have been previously described [23, 24] (Tables S3 and S4).

### Correlation with public datasets

Bulk RNA-seq data for head and neck squamous cell carcinoma (HNSCC) and normal or paraneoplastic samples were obtained from The Cancer Genome Atlas (TCGA) (https://portal.gdc.cancer.gov/), along with clinical and follow-up data for patients with HNSCC. The top 10 DEGs were considered marker genes for cell subsets. Marker genes for effector T cells, exhausted T cells, and Tregs were obtained from GEPIA2 [25]. Mean TPM levels of marker genes were log_2_ transformed and used as gene signatures. Spearman’s correlation analysis was performed to estimate relationships between specific cell types.

The online tool CIBERSORTx with default parameters [26] was used to create a reference signature matrix from our single-cell RNA-seq dataset and estimate the cell-type proportions for TCGA-HNSC cohort patients based on a constructed cell-type reference. Subsequently, cell subsets were divided into high- and low-infiltration groups based on the optimal cut-off value determined using the surv_cutpoint function from the Survminer R package. To evaluate the prognostic value of cell clusters, Kaplan–Meier analysis was performed using the survival R package.

### Immunohistochemistry

In brief, paraffin sections of HPSCC tumors were deparaffinized, hydrated, and antigen retrieval was performed at 100 °C in citrate buffer (pH 6.0) for 20 min. After cooling to 25 ℃, the sections were incubated with SPRR3 (Proteintech, 11742-1-AP), Ki67 (Origene, TA802544), LAMC2 (Abcam, ab210959), and fibroblast activation protein (FAP, Abcam, ab207178) antibodies overnight at 4 °C, followed by incubation with the goat anti-rabbit/mouse IgG-HRP polymer (Proteintech, PK10006) for 1 h at 25 ℃. Sections were then developed using a diaminobenzidine chromogenic solution, counterstained using hematoxylin, dehydrated using ethanol, cleaned with xylene, and mounted.

To quantify the target protein expression, the percentage of positive cells and the staining intensity were determined in five randomly selected fields using a microscope at 400 × magnification. For this, the cells were counted by two independent pathologists, neither of whom had knowledge or information regarding the clinical status of the patients. Scoring was conducted as previously described [27]; scores ≥ 2 points were considered positive expression.

To explore the relationship between the target protein and patient prognosis, survival analysis was performed in the R package “survival”, and the Survfit function was used to model the Kaplan–Meier survival curve.

### Multi-color immunohistochemistry (IHC)

Multi-color IHC assays were performed using multiplex immunohistochemistry (mIHC) staining kits (Absin, Shanghai, China), according to the manufacturer’s instructions. Immunofluorescence images were captured using TissueFAXS Spectra (TissueGnostics, Vienna, Austria). Antibodies used were FAP (BM5121, Boster, 1:250), PLA2G2A (K007485P, Solarbio, 1:100), panCK (GM351507, GeneTech, 1:1), α-SMA (19245, CST, 1:200), and SPP1 (ab214050, Abcam, 1:50).

### Flow cytometry analysis

Fresh tumor tissues and the corresponding adjacent tissues were harvested from patients with HPSCC and washed in PBS to remove the blood. Next, the tissues were cut into 2-mm pieces and digested using digestive enzymes [10% collagenase/hyaluronidase + 90% (DMEM + 5% FBS) + 1 mg/mL DNase I] at 37 °C on a shaking table (140 rpm) for 30 min, centrifuged at 300 × *g* at 4 °C for 5 min, and filtered with a 70-μm strainer to prepare a single-cell suspension.

Single-cell suspensions were resuspended in FACS buffer (PBS + 2% FBS) and incubated with anti-CD16/CD32 (BioLegend, 156603) for 20 min on ice. For cell membrane staining, the samples were first stained with Dead Cell Stain (Thermo Fisher, USA) for 15 min at 25 ℃ and then incubated with the cell surface markers CD4 (FITC), CD127 (APC), CD11b (PerCP/Cy5.5) from eBioscience, CD45 (Spark BlurTM550), CD3 (AF700), CD25 (PE/Cy7), PD-1 (PE/Dazzle594), CCR7 (Spark NIRTM685), CD11C (BV510), HLA-DR (BV421), and PD-L1(BV650) from Biolegend, and LAMP3 (PE, BD) for 30 min at 4 °C. Spectroscopic flow cytometry was performed for detection and analysis.

### Fibroblast culture and preparation of conditioned media

The cancer tissues and adjacent normal tissues of four patients with HPSCC were minced with sterile scissors, placed in 15 mL centrifuge tubes, and 2 mL of trypsin was added. The tissue was digested for 5 min, and then 6 mL of complete medium was added to terminate the digestion. After centrifugation (300 × *g* at y °C for 5 min), 2 ml of complete medium was added to resuspend the tissue, which were then transferred to a 10 cm culture dish, and place in a cell culture incubator (37 °C, 5% CO_2_, and 95% humidity). The next day, after the tissue had adhered to the culture dish wall, 5 mL of culture medium was added. Over time, fibroblasts migrate from the tissue onto the surface of the dish and are subcultured when the cells reached about 80% confluency. In this experiment, the third to fourth generation of cells in the logarithmic growth phase were used.

Fibroblasts were seeded on six-well culture plates (3 × 10^4^ cells/well) containing 2 mL of DMEM containing 10% FBS and cultured for 24 h. The medium was then replaced with serum-free DMEM, and cells were incubated for an additional 48 h. The supernatant was then collected, centrifuged at 1,000 × *g* at 4 °C for 5 min, and termed conditioned media (CM) of CAFs (CAF^CM^) and NFs (NF^CM^).

### Wound‐healing and transwell invasion assay

For the wound-healing migration assay, SUN1076 or FaDu cells were cultured for 24 h on six-well plates with DMEM containing 10% FBS and grown to 100% confluency. A scratch was made in each well using a pipette tip. Cells were washed twice with PBS and cultured in serum-free medium containing 25% medium without FBS, CAF^CM^, or NF^CM^ for 24–72 h. Images were captured at 0, 24, 48, and 72 h. Finally, ImageJ (National Institutes of Health, Bethesda, MD, USA) was used to measure migration distances.

For the transwell invasion assay, 1 × 10^5^ SUN1076 or FaDu cells were seeded on upper 24-well transwell chambers (Corning, USA, 3422) coated with 80 μL of Matrigel (BD Biosciences, USA; 356234) in serum-free DMEM. Medium containing 20% FBS was added to the lower chambers for 48 h to induce chemotaxis. Cells that migrated through the 8-μm pores were fixed with methanol and stained with 0.1% crystal violet. Stained cells were visualized using a microscope and those in five random fields were counted.

### Protein extraction and western blotting

SUN1076 or FaDu cells were cultured in DMEM containing 10% FBS for 24 h. When cells reached 60% confluency, they were washed twice with PBS and cultured in serum-free medium containing 25% CAF^CM^ or NF^CM^. After 48 h, cells were harvested in RIPA lysis buffer for protein extraction.

Protein concentration in cell lysates was quantified using a bicinchoninic acid assay. Protein samples (25 μg) were loaded onto a 10% gel, separated via SDS-PAGE, and transferred to a polyvinylidene difluoride membrane. The membrane was blocked with 5% milk diluted in Tris buffered saline containing 0.1% Tween 20 for 1.5 h, followed by overnight incubation with primary antibodies (human anti-E-cadherin, sc-8423; human anti-N-cadherin, sc-8424; human anti-Twist, sc-81417; human anti-vimentin, 10366-1-AP) at 4 °C. On the following day, the membrane was incubated with the secondary antibody (anti-rabbit-HRP, CST, 7074 s) for 1 h and visualized using an enhanced chemiluminescence reagent (Thermo Fisher, USA) and a Bio-Rad chemical exposure apparatus.

### Tube formation assay

Matrigel matrix (60 µL; BD Biosciences) diluted at a 1:1 ratio with DMEM was added to each well of a 96-well plate on ice and solidified at 37 °C. A HUVEC suspension (100 µL; 3 × 10^4^ cells/100 µL) was mixed and cultured on solidified Matrigel plugs in DMEM complete medium at 37 °C in a humidified atmosphere containing 5% CO_2_ for 4 h. Then, 100 µL of CAF^CM^ (experimental group), NF^CM^ (negative control), or DMEM (blank control) was added to the medium. Images were captured using a light microscope at 10 × magnification. Total branching length, number of nodes and junctions, and total mesh areas were calculated in ImageJ using the “Angiogenesis” plug-in, following a previously described protocol [28].

## Results

### Single-cell transcriptomic atlas and cell typing of HPSCC

**Fig. 1:**
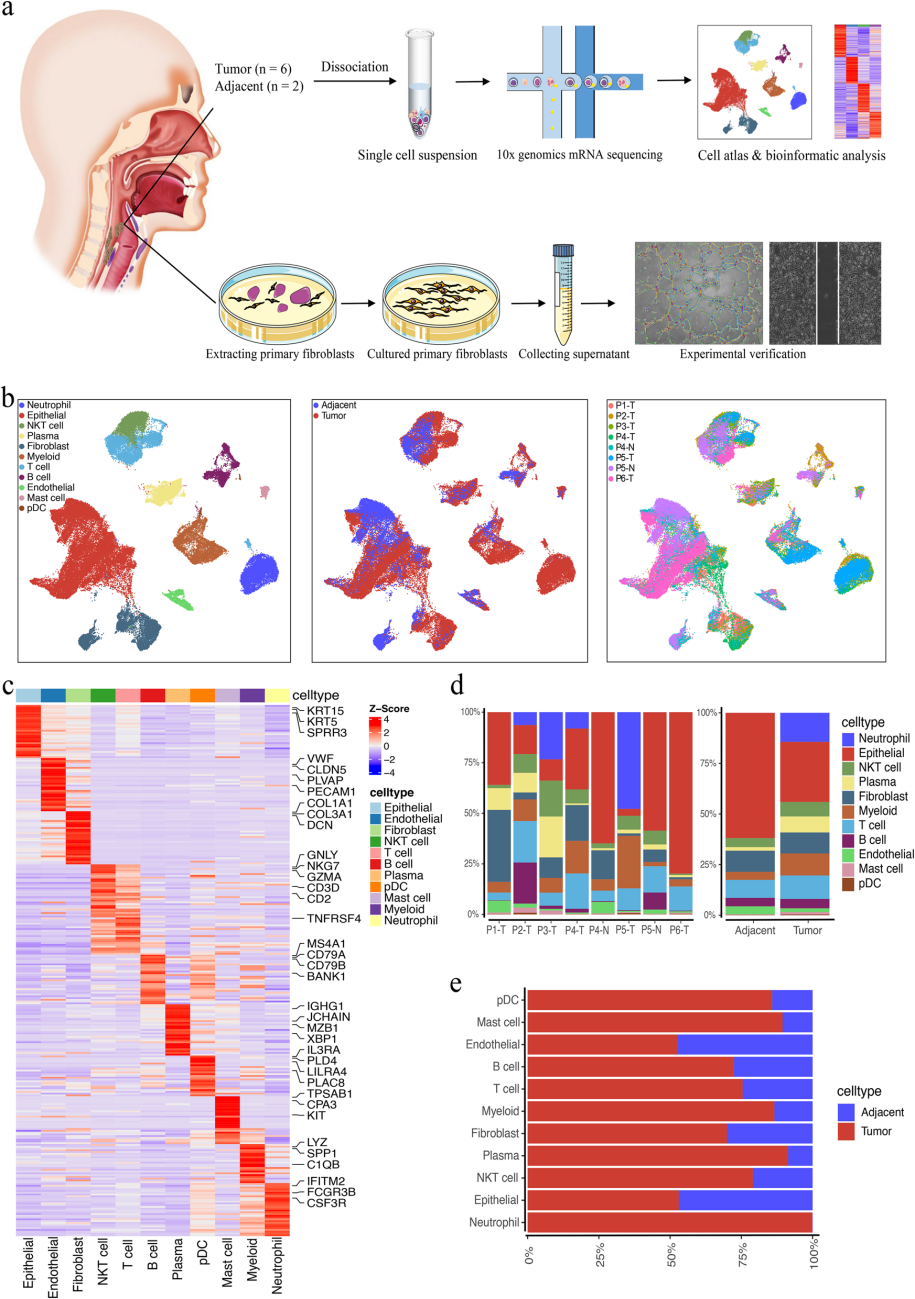
Single-cell transcriptomic landscape of HPSCC. **a** Schematic diagram of the experimental design and analysis. **b** UMAP plot of the clustering of 67,025 cells from all eight tumor and adjacent normal tissues samples, color coded by cell type (left), sample type (middle), or patient origin (right). **c** Heatmap showing signature DEGs between different cell types in HPSCC. **d** Bar plots showing the proportion of 11 major cell types for different donors (left) and tissues (right). **e** Frequency per cell type in tumor and adjacent normal tissue samples

To elucidate the cellular composition of HPSCC, we generated scRNA-seq profiles for six primary HPSCC tissues and two adjacent normal mucosae from six treatment-naïve patients (Fig. 1a, Table S1). After standard procedures, we acquired 67,025 cell transcriptomes for subsequent analysis. Adjacent nonmalignant tissues contributed 19,892 cells and tumors contributed 47,133 cells.

We identified 11 major cell types based on DEGs and canonical markers (Fig. 1b, c): EpCs (*n* = 26,735), endothelial cells (ECs; *n* = 1,583), fibroblasts (*n* = 6,866), NKT (natural killer T) cells (*n* = 4,343), T cells (*n* = 7,329), B cells (*n* = 3,075), plasmocytes (*n* = 4,126), plasmacytoid dendritic cells (pDCs; *n* = 166), mast cells (*n* = 617), myeloid cells (*n* = 5,331), and neutrophils (*n* = 6,854). These DEGs and marker genes confirmed the accuracy of cell identity, as shown in the Heatmap and Uniform Manifold Approximation and Projection (UMAP) plots (Fig. 1b, c, Fig. S1). Although all 11 cell types were present in both tumor and adjacent normal tissues, the proportion of each cell type varied greatly by sample, suggesting molecular intertumor heterogeneity (Fig. 1d, e).

### Transcriptomic intertumor heterogeneity of malignant EpCs and surrounding nonmalignant EpCs

**Fig. 2:**
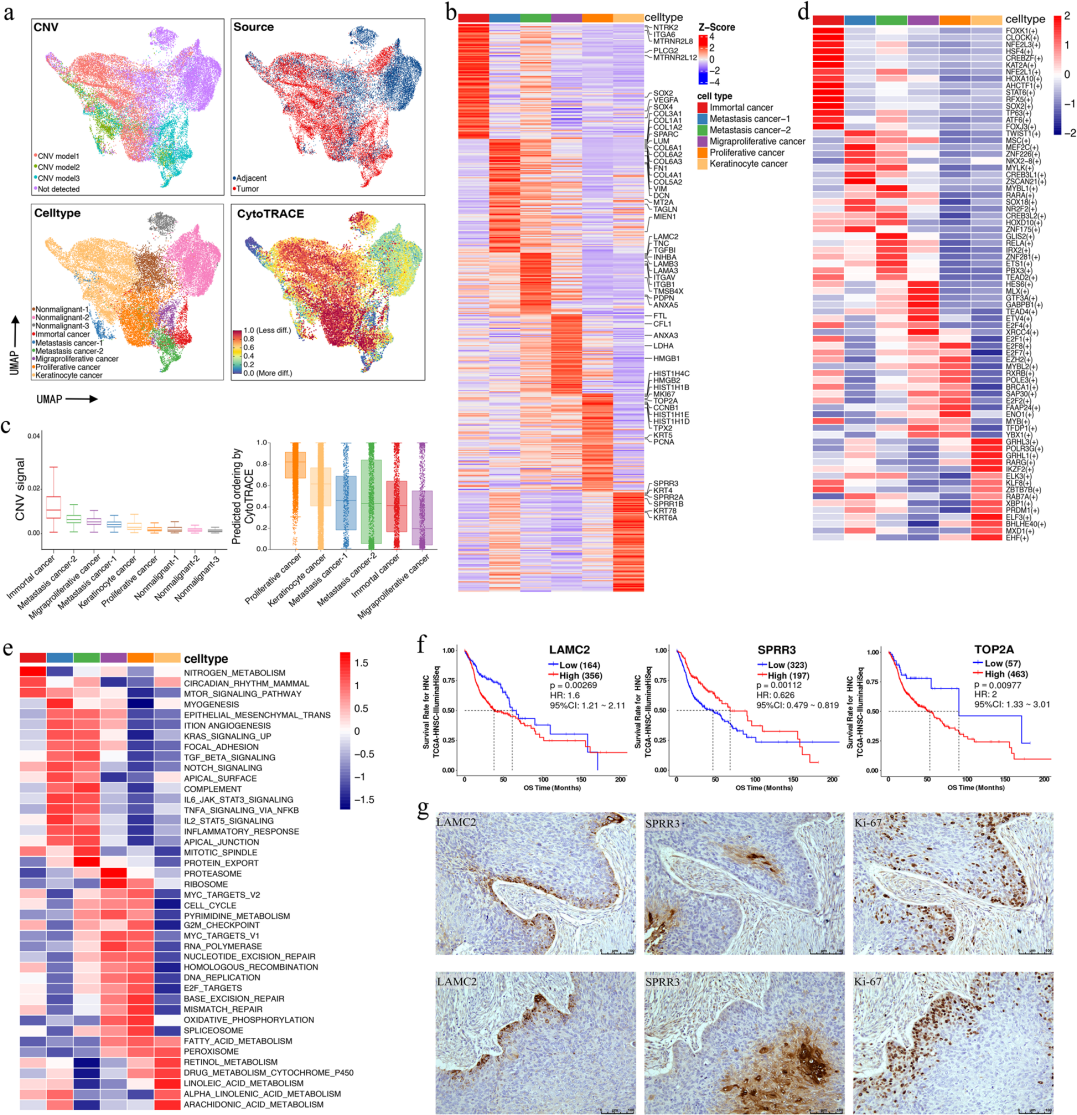
Transcriptomic heterogeneity and characterization of malignant epithelial cells in HPSCC. **a** UMAP plot of epithelial cells colored by the CNV level, sample type, cell type, and CytoTRACE score. **b** Heatmap showing the signature DEGs between the six distinct mEpC subsets. **c** Box plot depicting the CNV and CytoTRACE scores of the different epithelial cell subsets. **d** Heatmap of the *t*-values for the area under the curve (AUC) scores of expression regulation by the TFs of the mEpC subsets, as estimated using SCENIC. **e** Differences in the activities of hallmark pathways between different mEpCs subsets, scored using GSVA. **f** Kaplan–Meier curve of the OS in the TCGA-HNSC cohort stratified by the optimal cut-off point for LAMC2, SPRR3, and TOP2A expression levels. *P*-values were calculated using the two-sided log-rank test. **g** IHC staining showing the expression and localization of the p-EMT marker LAMC2, epithelial differentiation marker SPRR3, and proliferative marker Ki67 (MIK67) in the HPSCC tumor samples

We reclustered EpCs into 20 clusters. Although batch effects were removed, tumor cells still exhibited patient-specific expression patterns, indicating high intertumor heterogeneity (Fig. S2a). The InferCNV R package [17] detected the initial CNV per region in EpCs. If EpCs contained minimal or no CNVs, they were considered nonmalignant (nEpCs), while the remainder were considered malignant (mEpCs) (Fig. 2a, Fig. S2b). Tissue origin data for EpCs further supported the accuracy of CNV analysis in predicting EpC malignancy (Fig. 2a). Gain and loss patterns of DNA copy numbers varied across tumor cells; mEpCs could be divided into three types based on these patterns, indicating that tumor heterogeneity is partly caused by CNV variation (Fig. 2a; Fig. S2b).

However, CNV variation alone was insufficient for distinguishing mEpCs subclusters well (Fig. 2a). Therefore, to further explore EpC phenotypic characteristics in HPSCC, we subdivided mEpCs into six subsets based on the expression of canonical marker genes and the top DEGs in each cluster (Fig. 2a, b; Fig. S2c). CytoTRACE [18] analysis was performed to predict EpC differentiation states and identify quiescent stem cells in HPSCC (Fig. 2c). Specifically, immortal cancer cells had the highest CNV and high expression of immortal marker genes, such as *MTRNR2L8* and *MTRNR2L12*, indicating inhibition of apoptosis. Proliferative cancer cells highly expressed proliferation-related genes (*MKI67*, *TOP2A*, and *TPX2*); they also had the highest CytoTRACE score, reflecting strong proliferative potential and higher stemness characteristics. Keratinocyte cancer cells highly expressed epithelial differentiation marker genes (*SPRR3* and *SPRR2A*), while migratory proliferative (migraproliferative) cancer cells highly expressed malignancy-promoting factors *MARCKSL1*, *RPL39L*, *ANXA3*, and *HMGB1*. We identified two subsets with different epithelial-mesenchymal transition (EMT) characteristics. Metastatic cancer-1 cells highly expressed mesenchymal markers (e.g., *VIM*, *FN1*, and *COL1A1*), typical of EMT, whereas metastatic cancer-2 cells highly expressed partial EMT (p-EMT) signature genes (e.g., *LAMC2*, *PDPN*, and *INHBA*) [9], indicative of a p-EMT phenotype. RNA velocity analysis [20] and diffusion maps inferring cellular fate showed a directional flow from proliferative cancer cell to various mEpC types, supporting the stemness characteristic of this cell subset (Fig. S2d). Notably, CAFs are partially derived from mEpCs undergoing EMT [29]; we observed a strong directional flow of metastatic cancer-1 cells to CAFs, indicating that metastatic cancer-1 cells are a mEpC subset with complete EMT characteristics and metastatic cancer-2 cells undergoing p-EMT do not transition to the same extent (Fig. S2e).

Subsequently, we performed SCENIC analysis [21] to identify TFs regulating mEpC phenotypes and further confirm the accuracy of mEpC typing results (Fig. 2d; Fig. S2f). For instance, FOXK1, a specific TF of immortal cancer cells, plays a key role in regulating cell viability, proliferation, and life span [30]. TWIST1 is a TF in metastatic cancer-1 cells and an EMT driver. Proliferative cancer cells specifically express TFs involved in cell proliferation, including E2F TF family members (E2F2, E2F8, and E2F7) [31]. Lastly, GRHL3 is a surface epithelium commitment master regulator that plays a crucial role in keratinocyte cancer cell transcriptional regulation [32]. Furthermore, our inference was also supported by GSVA of biological pathways and characteristics in mEpC subsets (Fig. 2e). The pathways (such as EMT, angiogenesis, and focal adhesion pathways) related to tumor invasion and metastasis were enriched in metastasis cancer-1, metastasis cancer-2, and migratory proliferative cancer subsets. Metastasis cancer-1 and -2 subsets also shared common activated immune-related pathways (e.g., TGF-β, IL-6/JAK-STAT3, and TNF-α/NF-κB) that are closely related to EMT occurrence, tumor invasion, and metastasis [33]. Finally, as expected, proliferation (i.e., MYC targets v1 and v2, G2M checkpoint, E2F targets, RNA polymerase, and DNA replication) and DNA repair (i.e., mismatch and base excision repair) pathways, along with energy-producing oxidative phosphorylation, were enriched in migraproliferative cancer and proliferative cancer cell subsets.

To further explore the influence of different mEpCs subsets on the therapeutic effect and prognosis of patients, Kaplan–Meier survival analysis was performed on TCGA-HNSC cohort patients. The results indicated that higher expression of p-EMT, EMT, migraproliferative marker, and proliferation-related genes was associated with worse OS; conversely, expression of keratinocyte differentiation marker genes, such as *SPRR2A*, *SPRR3*, and *KRT78*, was associated with better OS (Fig. 2f; Fig. S2g–k).

Tumor cells exhibiting p-EMT rather than typical complete EMT characteristics can be more invasive because they respond to TME cues, increasing metastasis risk [34]. Therefore, we investigated the in situ spatial localization of cells expressing p-EMT within HPSCC tumors. The results further confirmed the accuracy of mEpC clustering and the variation in the spatial localization of different mEpC subsets (Fig. 2g). Metastatic cancer-2 cells co-stained for p-EMT markers (LAMC2) were localized at the tumor margin close to the surrounding stroma. Conversely, keratinocyte cancer cells stained with an epithelial differentiation marker (SPRR3) were located at the tumor core, consistent with the negative correlation between these programs suggested by the survival analysis. Interestingly, the Ki-67 protein (MKI67)-expressing proliferative cancer cells were located at the tumor margin between LAMC2- and SPRR3-positive cells, further supporting the proliferative potential to differentiate into various mEpC types.

### mCAFs promote cancer cell invasion and are associated with HPSCC progression

Fibroblasts are the predominant cells in stroma and contribute to tumorigenesis and tumor progression [35]. However, the definition of fibroblast subtypes and CAFs in HNSCC is controversial. We explored stromal fibroblast heterogeneity and characteristics in HPSCC to understand their effects on mEpC biological functions and their role in TME remodeling.

**Fig. 3:**
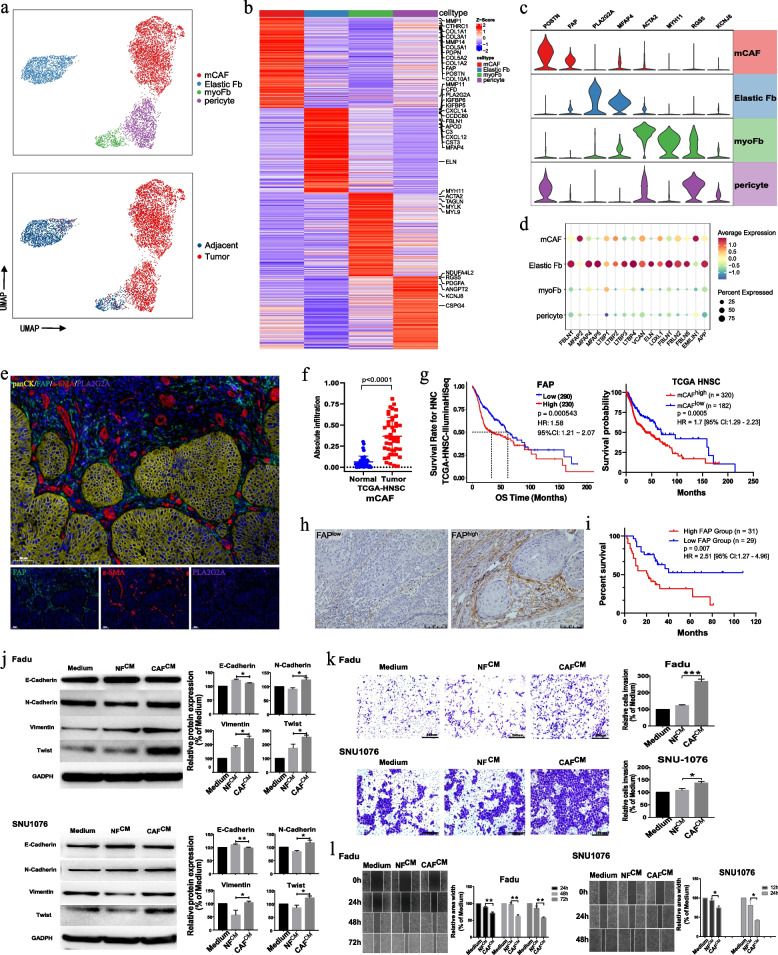
Fibroblast subsets in HPSCC tumor and adjacent normal tissues. **a** UMAP plot of fibroblast cells colored by cell and sample type. **b** Heatmap showing signature DEGs among four fibroblast subsets. **c** Violin plots showing marker gene expression in fibroblast subsets. **d** Bubble heatmap showing marker gene expression in Elastic Fbs. Dot size indicates fraction of expressing cells, colored according to expression normalized based on z-scores. **e** Representative images showing mIHC staining of panCK, FAP, ⍶-SMA, and PLA2G2A in HPSCC samples, in individual and merged channels. Scale bar represents 50 μm. **f** Absolute infiltration proportion of mCAFs comparing normal (*n* = 43) and tumor tissues (*n* = 43) in the TCGA-HNSC cohort. **g** Kaplan–Meier curve of the OS in the TCGA-HNSC cohort stratified by the optimal cut-off for FAP expression and mCAF infiltration. **h** Representative images of the IHC staining of HPSCC tumor samples with a high and low FAP expression. **i** Comparing OS (Kaplan–Meier curves) among patients with HPSCC who either lowly or highly express FAP. **j** FaDu or SNU1076 cells were incubated for 48 h with normal and CM of NFs and CAFs; E-cadherin, N-cadherin, vimentin, Twist, and GADPH expression was evaluated using immunoblotting. Cropped blots are used here and the full-length gel images are available in Additional file 3 (Fig. S7). **k** FaDu or SNU1076 cell invasion in CM relative to complete growth medium measured after 48 h. Photographs are representative of randomly chosen fields. **l** Representative images showing wound healing of FaDu or SNU1076 cells in CM of NFs and CAFs relative to complete growth medium, at 0, 12, 24, 48, and 72 h after wound infliction. Significance in (**g**) and (**i**) was assessed with two-sided log-rank tests. In (**j**–**l**), data are shown as mean ± SEM, with *n* = 3 paracancerous tissues and n = 4 tumor tissue columns. Differences were determined using unpaired *t*-tests (*P < 0.05; **P < 0.01; ***P < 0.001)

Fibroblasts were divided into nine clusters (Fig. S3a). Clusters 0, 2, 3, and 8 were mainly derived from tumor tissues and characterized by high expression of classical CAF markers (*FAP*, *PDPN*, and metalloproteinases) and extracellular matrix (ECM) signature proteins such as multi-collagen molecules and periostin (*POSTN*); therefore, we defined these clusters as mCAFs (Fig. 3a–c). Clusters 4 and 5 mainly derived from tumor tissues and were designated as pericytes due to their high expression of *RGS5* and *KCNJ8* (Fig. 3a–c). Cluster 6 mainly derived from adjacent tissues and showed an upregulation of canonical myofibroblastic markers, including *ACTA2* and genes for contractile proteins (*TAGLN*, *MYLK*, *MYL9*, and *MYL11*); therefore, we designated this group as myofibroblasts (myoFbs; Fig. 3a–c). Clusters 1 and 7 expressed elastic fiber differentiation genes (Fig. 3d), especially tropoelastin (*ELN*), fibrillin-1 (*FBLN1*), and microfibril associated protein 4 (*MFAP4*); these were termed elastic fibroblasts (Elastic Fbs; Fig. 3a–d) [8]. The presence of major fibroblast subsets in HPSCC samples was confirmed with mIHC staining (Fig. 3e).

**Fig. 4:**
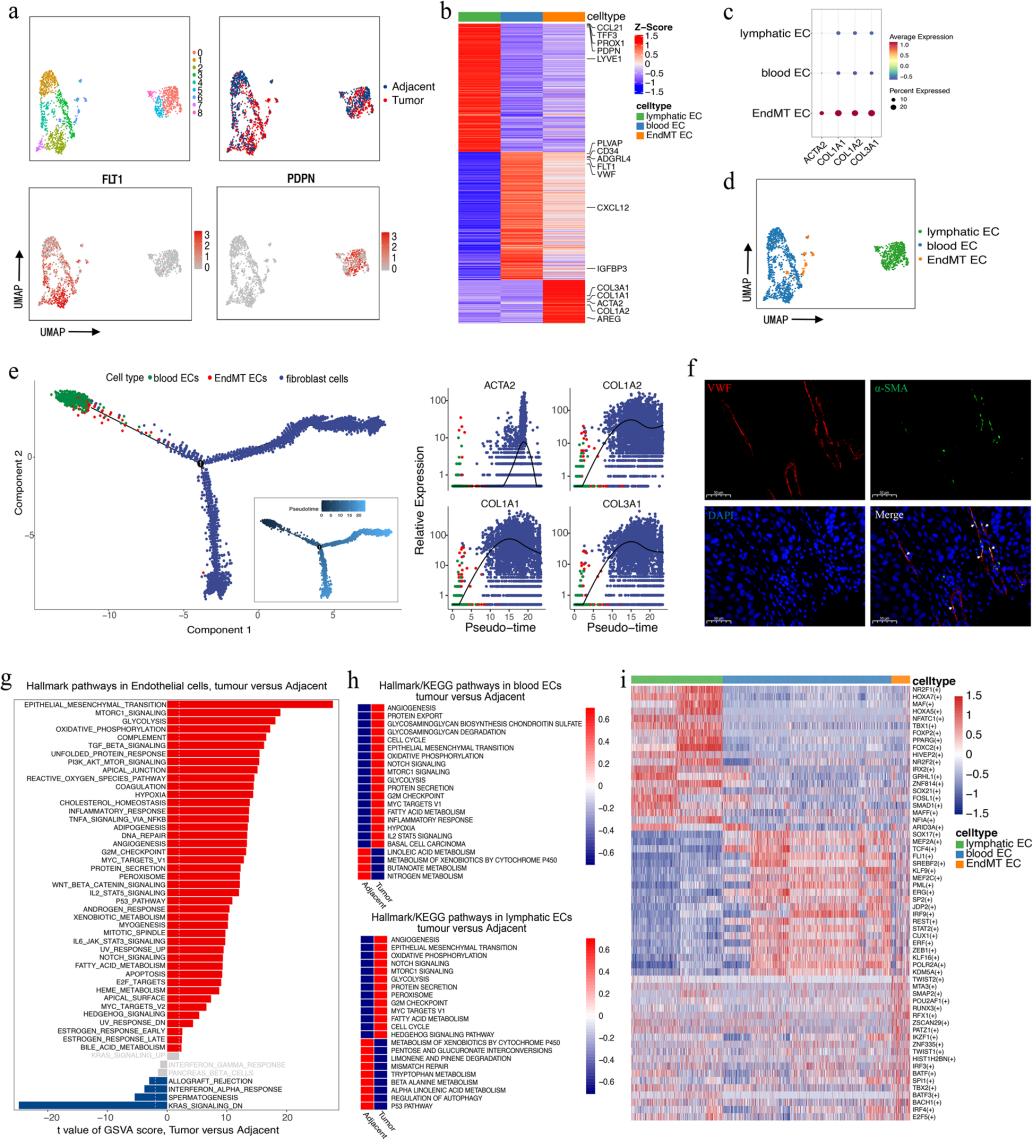
Detailed characterization of ECs in HPSCC. **a** UMAP plot of ECs colored by cluster, sample type (up), and subgroup markers (down). **b** Heatmap showing signature DEGs between three distinct EC subsets. **c** Bubble heatmap showing the ACTA2, COL1A1, COL1A2, and COL3A1 expression among the different EC subsets. Dot size indicates the fraction of expressing cells, colored according to the z-score normalized expression levels. **d** UMAP plot of ECs colored by cell type. **e** Pseudotime trajectory analysis of ECs and fibroblast cells. Each dot represents one single cell, colored according to its cluster label. The inlet plot shows each cell with a pseudotime score from dark blue to light blue, indicating an early and terminal state, respectively. A jitter plot showing expression changes in ACTA2, COL1A1, COL1A2, and COL3A1 over pseudotime. **f** Representative images showing mIHC staining of VWF and α-SMA in HPSCC tumor samples in individual and merged channels. Scale bar = 50 μm. **g** Differential pathways enriched in tumor and normal ECs according to GSVA score (two-sided unpaired limma-moderated *t*-test). **h** Differences in pathway activities scored per cell using GSVA between blood and lymphatic ECs in tumor versus normal tissues. **i** Heatmap representing t-values for AUC scores of gene regulation based on transcription factor expression in EC subsets, estimated using SCENIC

Pericytes play a crucial role in tumor neovascularization [36]; consistently, we observed a strong angiogenic signature (such as for *ANGPT2*, *CAV1*, *PDGFA*, *THY1*, *EGFL6*, and *GMFG*) in pericytes (Fig. S3b). Moreover, we observed a significant increase in the pericyte fraction of tumor samples when CIBERSORTx [26] was used to estimate cell abundance from the TCGA-HNSC dataset (*P* = 0.0001; Fig. 4c). Patients with a higher pericyte infiltration level had remarkably worse prognoses (Fig. S3c).

The scRNA-seq data and mIHC staining results indicated that mCAFs were the predominant CAF subset in the HPSCC tumor tissues (Fig. 3a; Fig. S3d); furthermore, significantly enriched mCAFs in HNSCC samples were observed in the TCGA-HNSC cohort (Fig. 3f). Notably, mCAFs highly expressed tumor invasion-associated genes (*FAP*, *MMP1*, *MMP14*, and *POSTN*; Fig. 3b), commonly associated with ECM remodeling and cell migration during tumor metastasis, thus reflecting the importance of mCAFs in the metastatic TME. Analysis of the TCGA-HNSC cohort data revealed that patients with high FAP expression had poor OS (Fig. 3g). More importantly, consistent with mCAF characteristics, the TCGA-HNSC cohort patients with higher mCAF infiltration had poor OS (Fig. 3g). These results were verified through IHC staining for a mCAF marker (FAP) of 60 paraffin-embedded HPSCC tumor samples from our own specimen library (Fig. 3h). Consistent with the TCGA results, the prognostic analysis confirmed that high mCAF marker (FAP) expression in the stroma was associated with a poor OS (HR: 2.51, 95% confidence interval: 1.27–4.96, *P* = 0.007; Fig. 3i). IHC/mIHC staining also showed that FAP-positive fibroblasts (mCAFs) were located near the tumor nest edge and in close contact with p-EMT cells there, prompting consideration of the interaction between mCAF and mEpCs, such as ligand-receptor signaling (Fig. 3h; Fig. S3e). Subsequently, CellphoneDB2 cell communication analysis [37] confirmed that mCAFs interacted more strongly with mEpCs than with other fibroblasts in tumor samples. In particular, the interaction between metastatic cancer-2 cells and mCAFs was the most frequent, and the ligand-receptor pairs were enriched in various cancer-related pathways, including the Notch, Wnt, and TGF-β pathways (Fig. S4). Furthermore, GSVA enrichment revealed pathways that were significantly enriched in mCAFs during EMT, including the TGF-β, IL-6/JAK-STAT3, and TNF-α/NF-κB signaling pathways (Fig. S3f). These results indicate that mCAFs can remodel the ECM and interact with tumor cells to promote the EMT program, leading to tumor metastasis.

Next, we isolated fibroblasts from HPSCC tissues to investigate the biological characteristics of mCAFs. Co-culture of HNSCC cells (FaDu and SNU1076) with the CM of NFs and CAFs (NF^CM^ and CAF^CM^) (Fig. S3g) revealed that EMT markers, such as N-cadherin and vimentin, were upregulated in the tumor EpCs of the CAF^CM^ group compared with those in the NF^CM^ group, whereas epithelial biomarkers, such as E-cadherin, were downregulated (Fig. 3j). Furthermore, Twist expression was upregulated the most in the CAF^CM^ group; thus, Twist overexpression might induce EMT in HNSCC. Moreover, the CAF^CM^ group had significantly higher tumor cell migration and invasion capacity than the NF^CM^ and control groups (Fig. 3k, l), suggesting that CAF^CM^ activates the EMT program of HNSCC cells and promotes tumor invasion and metastasis.

SCENIC analysis revealed that the fibroblast subsets can be distinguished by different TF groups. Notably, mCAFs highly express both the EMT driver TWIST2 and angiogenic TFs (such as HIF1A, RUNX3, and FOXQ1; Fig. S3h, i). This result concurs with previous findings that hypoxia can promote angiogenesis in the TME by reprogramming CAFs [38] and agrees with the GSVA enrichment results indicating that mCAFs are enriched in VEGF signaling and angiogenesis pathways (Fig. S3f). Therefore, to further elucidate the interaction between CAFs and ECs and the angiogenesis-promoting CAF mechanism, we investigated the EC subsets.

### Tumor-associated ECs exhibit endothelial-to-mesenchymal transition signatures and high angiogenic activity

Detected ECs were further split into nine clusters to explore EC heterogeneity (Fig. 4a). They were then identified as blood or lymphatic ECs (marked with *FLT1* and *PDPN*, respectively). The ECs in Cluster 6 overexpressed both blood EC marker genes (*FLT1* and *VWF*) and fibroblast marker genes (*ACTA2*, *COL1A1*, *COL1A2*, and *COL3A1*), suggesting that these ECs underwent endothelial-mesenchymal transition (EndMT) [39]; therefore, we named this cell group as EndMT ECs (Fig. 4b–d). ECs undergoing EndMT are highly proliferative and invasive, crucial for tumor progression, and an important CAF source [40]. To further elucidate the EndMT program occurring in ECs in HPSCC and investigate the transition between ECs and CAFs, we performed EC and CAF pseudotime trajectory analysis [19]. These results are consistent with the above speculation, showing that blood ECs were located at the origin of the differentiation trajectory and that ECs undergoing EndMT were located in the intermediate stage on the pseudotime trajectory between blood ECs and CAFs. Moreover, *ACTA2*, *COL1A1*, *COL1A2*, and *COL3A1* expression gradually increased with the transition of EndMT EC to CAF (Fig. 4e). Multiplex IHC staining further verified the existence of EndMT ECs co-expressing VWF and α-SMA in tumor tissues (Fig. 4f).

The comparison of hallmark pathways between tumor and normal ECs via GSVA revealed that EMT was the top enriched signature in tumor ECs. Additionally, mTORC1 signaling, glycolysis, TGF‐β signaling, PI3K/AKT/mTORC signaling, hypoxia, TNF-α signaling via NF-κB, and angiogenesis were enriched in tumor ECs, suggesting the involvement of EndMT ECs in angiogenesis induction in HPSCC (Fig. 4g) [41–43]. Similarly, by separately comparing the hallmark pathways of blood and lymphatic ECs in tumor and adjacent tissues, we determined that angiogenesis was the most enriched feature in tumor ECs (Fig. 4h). Finally, SCENIC analysis identified TWIST2, MTA3, and SMAP2 as possible key EndMT EC differentiation-regulating TFs (Fig. 4i), among which TWIST2 has been confirmed as the key TF in EndMT ECs in brain arteriovenous malformations [44].

**Fig. 5:**
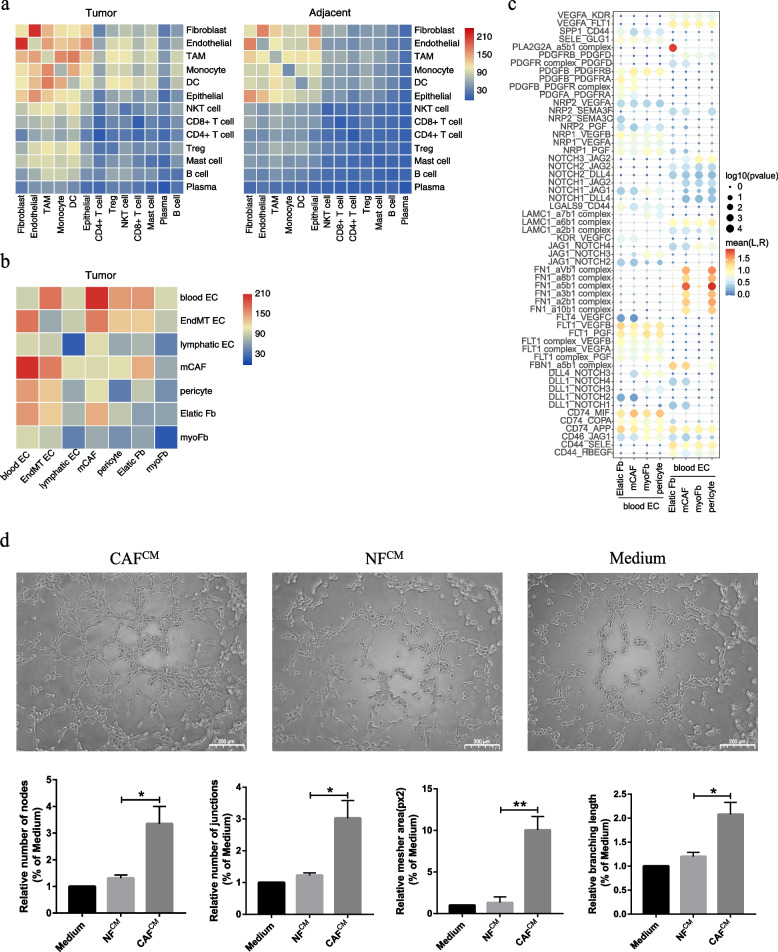
Cell–cell interactions in HPSCC and pro-angiogenic ability of mCAFs on HUVECs. **a** Heatmap representing the number of predicted ligand–receptor pairs between different cell types in tumor and normal samples. **b** Heatmap representing the number of predicted ligand and receptor pairs between different subsets of ECs and fibroblasts in tumor samples. **c** Dot plot of the predicted ligand–receptor interactions between different subsets of ECs and fibroblasts in tumor samples. **d** Representative images of the tube formation capability of HUVECs cultured for 4 h in normal and conditioned media of NFs and CAFs. Scale bar = 200 μm. Data of NF^CM^ and CAF^CM^ groups versus medium group are shown as mean ± SEM, with n = 3 paracancerous tissues and n = 4 tumor tissue columns. Differences were determined using unpaired *t*-tests (*P < 0.05; **P < 0.01)

CellphoneDB2 analysis revealed that ECs interacted most frequently with fibroblasts, and this interaction was more significant in tumor samples than in normal samples (Fig. 5a). When examining the cell–cell interactions of different EC and fibroblast subsets, blood ECs strongly interacted with the fibroblast subsets, especially mCAFs (Fig. 5b). The blood EC–fibroblast subset interaction is mainly mediated by PGF, VEGFA, and PDGF and their corresponding protein receptors, which are known angiogenesis drivers (Fig. 5c). The tight interaction between ECs and mCAFs in the TME suggests the close involvement of mCAFs in tumor angiogenesis and vasculature maintenance. As expected, the tube formation assay showed that CAF^CM^ significantly accelerated tube formation compared to the medium (used as the control group) and NF^CM^, suggesting the angiogenesis-promoting potential of CAFs (Fig. 5d).

### SPP1^+^ macrophages are associated with HPSCC progression and synergize with mCAFs to promote tumor progression

**Fig. 6:**
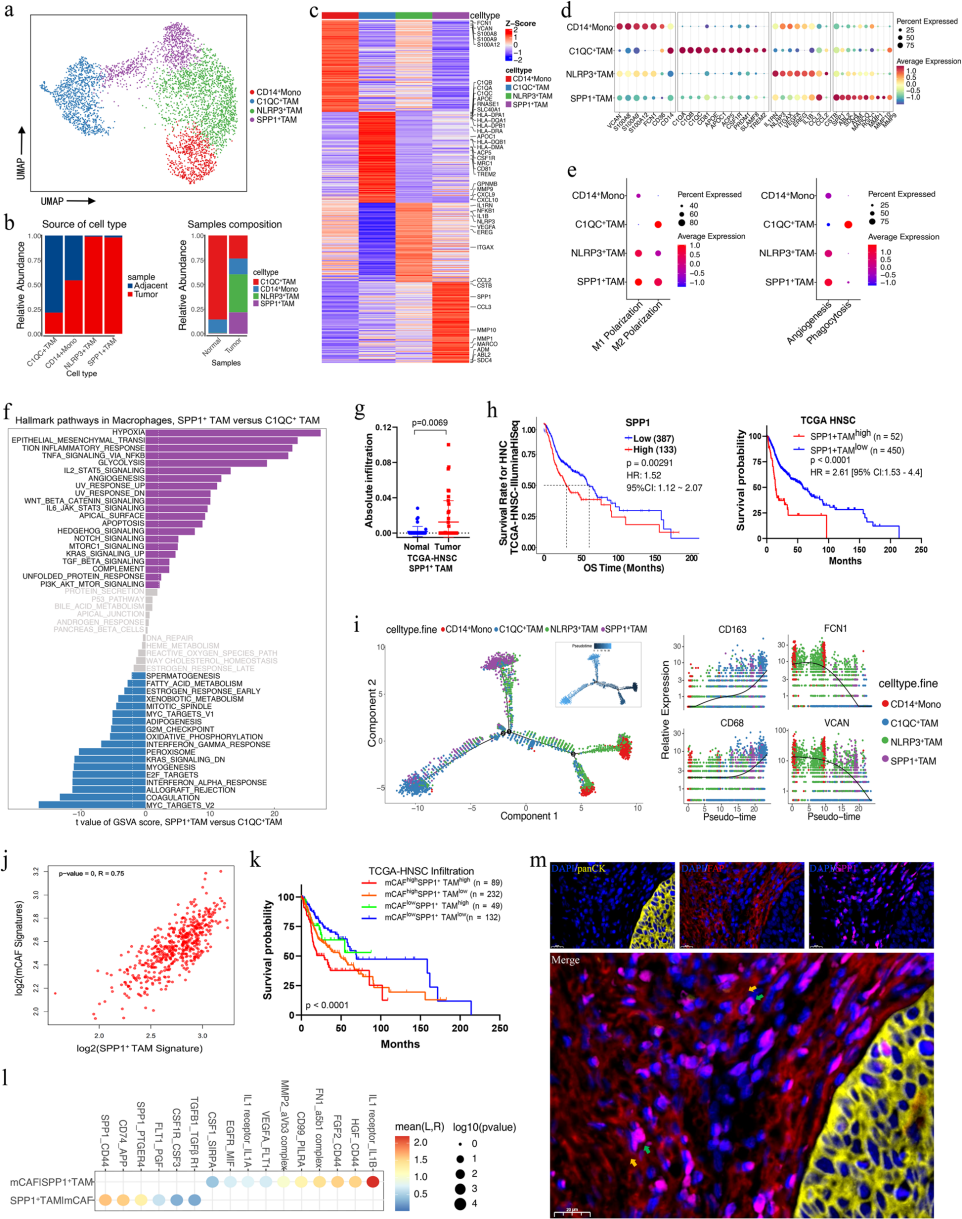
Detailed characterization of monocytes/macrophages in HPSCC. **a** UMAP plot of monocyte/macrophage cells colored by cell type. **b** Frequency (left) and proportion (right) of four major mononuclear/macrophage cell types in tumor and normal tissue samples. **c** Heatmap showing signature DEGs between mononuclear/macrophage cell types. **d** Bubble heatmap showing marker genes across mononuclear/macrophage cell types. Dot size indicates fraction of expressing cells, colored according to expression normalized to z-score. **e** Dot plot of representative M1, M2, angiogenic, and phagocytic signatures in monocyte/macrophage clusters [Z-score normalized log_2_ (count + 1)]. **f** Differential pathways enriched in C1QC^+^ and SPP1^+^ TAMs according to GSVA. Two-sided unpaired limma-moderated *t*-test. **g** Absolute infiltration proportion of SPP1^+^ TAMs compared between normal (*n* = 43) and tumor (*n* = 43) tissues in the TCGA-HNSC cohort. **h** Kaplan–Meier curve of OS in the TCGA-HNSC cohort stratified by optimal cut-off point for SPP1 expression and SPP1^+^ TAM infiltration. **i** Pseudotime trajectory analysis of mononuclear/macrophage cells. Each dot represents one cell, colored according to its cluster label. Inlet plot showed each cell with a pseudotime score from dark blue (early state) to light blue (terminal). Jitter plot showing expression changes in macrophage differentiation-associated genes over pseudotime. **j** Correlation of mCAF signature with SPP1^+^ TAMs based on TCGA-HNSC data. Each dot represents a patient (Pearson’s correlations). **k** Kaplan–Meier OS analyses of four subgroups in the TCGA-HNSC cohort, stratified by infiltration of both mCAFs and SPP1^+^ TAMs. **l** Dot plot of predicted ligand*–*receptor interactions between mCAFs and SPP1^+^ TAMs in tumor samples. **m** Representative images showing mIHC staining of panCK, FAP, and SPP1 in HPSCC tumor samples, in individual and merged channels. Scale bar = 20 μm. Significance in (**h**) and (**k**) was determined with two-sided log-rank tests

Myeloid cells were reclustered into monocytes/macrophages and DCs according to marker gene expression (Fig. S5a) and monocytes/macrophages were further identified as one monocyte type and three macrophage types (Fig. 6a); among these, three macrophage types were abundant in tumor tissues and designated as tumor-associated macrophages (TAMs; Fig. 6b). Specifically, CD14^+^ monocytes were identified based on the high expression of monocyte-related genes, such as *S100A8*, *S100A9*, *VCAN*, and *FCN1*; C1QC^+^ TAMs were characterized by high expression of multiple complement C1Q and antigen-presenting genes; NLRP3^+^ TAMs were characterized by *NLRP3* expression and the highest expression of *VEGFA* and *IL1B*; and SPP1^+^ TAMs showed high expression of *SPP1* and the scavenger receptor *MARCO* (Fig. 6c, d). Further comparison of the DEGs between the TAM subsets revealed that in addition to complement C1Q and antigen presentation genes, C1QC^+^ TAMs highly expressed immune activation-related genes, such as *CXCL9* and *CXCL10* (Fig. 6c), which are important for T lymphocyte recruitment and activation [45], thus suggesting that C1QC^+^ TAMs benefit HPSCC treatment. Conversely, SPP1^+^ TAMs expressed multiple pro-angiogenic/tumorigenic (*SPP1* and *MARCO*) and pro-proliferation, invasion, and migration genes (*CSTB*, *ABL2*, *SCD4*, and *ADM*; Fig. 6c, d) [46–50].

The results of the gene signature score showed that CD14+ monocytes almost did not express the M1 and M2 gene signatures, consistent with our definition (Fig. 6e). Notably, similar to previous studies, we found that the M1 and M2 signatures do not exist independently; instead, they are co-expressed in NLRP3^+^ and SPP1^+^ TAMs (Fig. 6e) [23, 51]. However, C1QC+ TAMs showed higher M2 instead of M1 signatures. These results suggest that the TAM phenotypes in the HPSCC TME are far more complex than those of the simple in vitro M1/M2 polarization model. Moreover, the angiogenic score of NLRP3^+^ TAMs, especially that of SPP1^+^ TAMs, was extremely high, while the phagocytic score of C1QC^+^ TAMs was the highest (Fig. 6e).

These results were validated using gene set enrichment analysis and GSVA, revealing that the antigen processing and presentation pathway for antitumor effects was significantly enriched in C1QC^+^ TAMs (Fig. S5b). Additionally, cancer-related pathways such as hypoxia, EMT, glycolysis, and angiogenesis were strongly enriched in SPP1^+^ TAMs. Thus, SPP1^+^ TAMs may be closely related to malignant HPSCC progression (Fig. 6f). the TCGA-HNSC cohort data indicated that the infiltration of SPP1^+^ TAMs was significantly higher in tumor samples than in the adjacent normal tissues, consistent with our scRNA-seq data (Fig. 6g). Furthermore, survival analysis verified that the TCGA-HNSC cohort patients with higher SPP1 expression or SPP1^+^ TAM infiltration levels had poorer prognoses and significantly poorer OS (Fig. 6h).

Next, pseudotime trajectory analysis revealed the relationship between monocyte-macrophage differentiation. The trajectory origin was determined using the expression of macrophage-differentiation signature genes over pseudotime. The analysis revealed that CD14^+^ monocytes were located at the origin of the differentiation trajectory, whereas TAMs were mainly enriched in the middle and differentiated ends, among which C1QC^+^ and SPP1^+^ TAMs were concentrated at the ends of different trajectory branches on both sides, indicating that C1QC^+^ and SPP1^+^ TAMs have completely different functional phenotypes and differentiation trajectories. Interestingly, the NLRP3^+^ TAM trajectories were distributed between the origin and SPP1^+^ TAMs, and some NLRP3^+^ and SPP1^+^ TAMs shared the same differentiation ends. By combining these results with that for the functional phenotypes, it can be inferred that NLRP3^+^ TAMs may be transformed into SPP1^+^ TAMs in the HPSCC TME (Fig. 6i).

Both mCAF and SPP1^+^ TAMs were mainly enriched in tumor tissues, and in many identical cancer hallmarks, such as EMT, angiogenesis, TGF-β, IL-6/JAK-STAT3, and TNF-α/NF-κB signaling pathways. To further reveal the relationship between mCAFs and SPP1^+^ TAMs, correlation analysis of the TCGA-HNSC cohort was performed, and the results of the mCAF and SPP1^+^ TAM signatures showed a strong positive correlation (Fig. 6j; *R* = 0.75, *P* < 0.0001, Pearson’s correlation); moreover, the patients with higher mCAF and SPP1^+^ TAM infiltration levels had shorter OS (Fig. 6k), suggesting that these two cell types synergistically promote tumor progression. Subsequently, cell communication analysis revealed key mediators of the interaction between mCAFs and SPP1^+^ TAMs, showing that SPP1^+^ TAMs specifically interact with mCAFs through SPP1_CD44 and CD74_APP (Fig. 6l) [52]. Moreover, SPP1^+^ TAMs promote mCAF activation by secreting cytokine signals encoded by *IL1B* or *TGFB1*, which in turn, can promote the formation of immunosuppressive microenvironment [53]. Finally, mIHC staining revealed that SPP1- and FAP-positive cells are physically juxtaposed in HPSCC tissues, corroborating the interaction between these two cell types (Fig. 6m). These results suggest that mCAF and SPP1^+^ TAMs may synergistically participate in TME remodeling and promote tumor angiogenesis and progression.

### LAMP3^+^ DCs display a tolerogenic phenotype in HPSCC

**Fig. 7:**
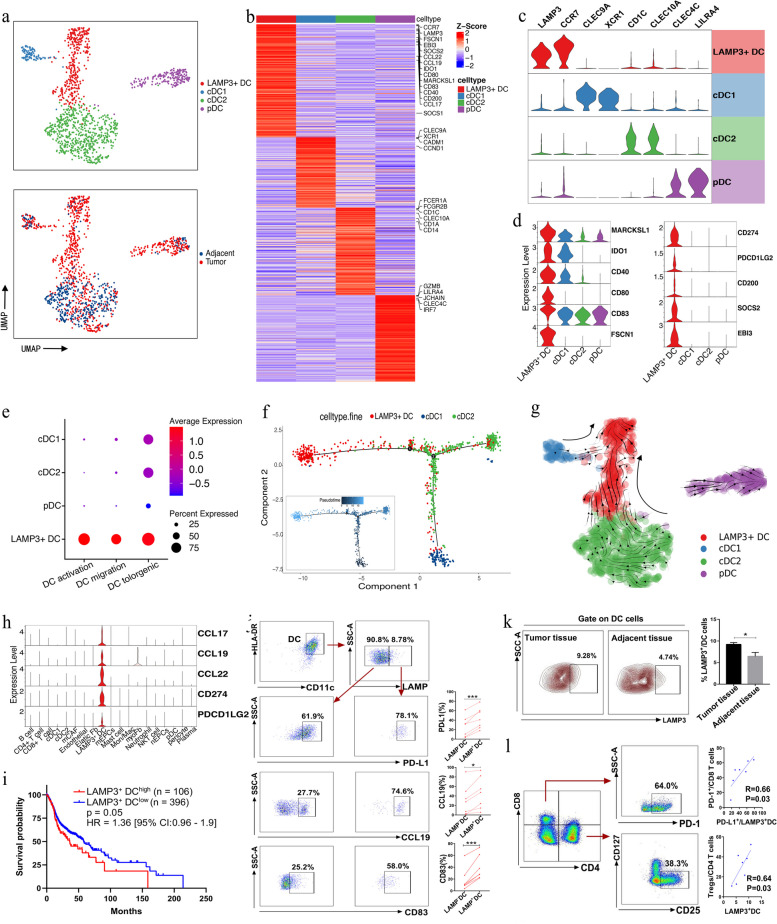
Detailed characterization of DCs in HPSCC. **a** UMAP plot of the DCs colored by cell (up) and sample (down) types. **b** Heatmap showing the signature DEGs among four distinct DC subsets. **c** Violin plots showing the expression of marker genes in the DC subsets. **d** Violin plot showing the expression of immune-suppressive genes in four distinct DC subsets. **e** Dot plot representative of the activation, migration, and tolerogenic signatures of DCs [Z-score normalized log_2_ (count + 1)]. **f** Pseudotime trajectory analysis of DCs. Each dot represents one single cell, colored according to its cluster label. The inlet plot shows each cell with a pseudotime score from dark blue to light blue, indicating an early and terminal state, respectively. **g** RNA velocities are visualized on the UMAP projection of DCs using Gaussian smoothing on a regular grid. **h** Violin plot showing the expression of the lymphocyte recycling chemokines CD274 and PDCD1LG2 in the major HPSCC cell types. **i** Kaplan–Meier curve of the OS in the TCGA-HNSC cohort stratified by the optimal cut-off point for the LAMP3^+^ DC infiltration level. *P*-values were calculated using the two-sided log-rank test. **j** Expression of PD-L1, CCL19, and CD83 on LAMP3^−^DCs or LMAP^+^DCs in tumor tissues (*n* = 7) was analyzed using flow cytometry. **k** Flow cytometry of LAMP3^+^ DCs infiltrated in the tumor and corresponding adjacent tissues (*n* = 4). **l** Correlation analysis of the PD-L1^+^LAMP3^+^ DCs and PD-1^+^CD8 T cells, LAMP3^+^ DCs, and Tregs infiltrated in the HNSCC tumor tissue (*n* = 7) using Spearman rank. R: correlation coefficient. Bars and errors are represented as mean ± SEM; data were analyzed using unpaired or paired t-tests (*P < 0.05; **P < 0.01, ***P < 0.001)

The DCs of myeloid cells and the pDC subset identified during the first clustering were subjected to unsupervised clustering to generate a DC map in HPSCC (Fig. 7a). Collectively, based on the marker gene expression, distinct conventional DC (cDC) and pDC subsets were identified, namely cDC1, cDC2, LAMP3 + DCs, and pDCs (Fig. 7b, c). Among these, LAMP3^+^ DCs were predominantly derived from tumors (Fig. 7a; Fig. S6a) and expressed the maturation markers *LAMP3*, *MARCKSL1*, and *IDO1*; the immune activation markers *CD40*, *CD80*, and *CD83*; and the migration markers *CCR7* and *FSCN1*, suggesting their high maturation, activation, and migration potential in HPSCC tumors (Fig. 7c, d). We then scored the DC subset activation and migration abilities and, expectedly, LAMP3^+^ DCs scored highest (Fig. 7e). Furthermore, LAMP3^+^ DCs had the highest tolerance score and exhibited a tolerogenic signature (Fig. 7e), consistent with high expression of immunosuppressive genes, including *IDO1*, *CD274* (*PD-L1*), *PDCD1LG2* (*PD-L2*), *SOCS2*, *EBI3*, and *CD200* (Fig. 7d). Subsequently, we further investigated the origin of LAMP3^+^ DCs and their lineage relationships with other DC subsets, pseudotime trajectory analysis revealed that cDC1 and cDC2 branches developed into LAMP3^+^ DCs, and LAMP3^+^ DCs had the highest pseudotime score, being the most differentiated and mature cells (Fig. 7f). Consistently, RNA velocity analysis predicted a transformation trend of cDC1 and cDC2 into LAMP3^+^ DCs (Fig. 7g).

LAMP3 + DCs express various genes encoding lymphocyte recirculation chemokines, including CCL17, CCL19, and CCL22, and it is known that CCL17 and CCL22 can form the CCL17/CCL22-CCR4 axis to recruit Tregs that express CCR4 [54]. The genes encoding these chemokines were almost entirely expressed by LAMP3^+^ DCs in HPSCC (Fig. 7h). To further reveal the relationship between LAMP3^+^ DCs and T cells, we applied correlation analysis to the TCGA-HNSC cohort and discovered that the LAMP3^+^ DC signature demonstrated a strong positive correlation with the Treg (*R* = 0.78, *P* < 0.0001, Pearson’s correlation) and exhausted CD8+ T cell signatures (*R* = 0.78, *P* < 0.0001) but not with the effector T cell signature (*R* = 0.37, *P* < 0.0001; Fig. S6b). Additionally, the levels of CD274 (PD-L1) and PDCD1LG2 (PD-L2) in LAMP3^+^ DCs were the highest among the cell subsets in HPSCC (Fig. 7i). These results reveal that LAMP3^+^ DCs are associated with T cell dysfunction; specifically, LAMP3^+^ DCs can inhibit CD8^+^ T cell function in the TME via the CD274/PDCD1LG2-PDCD1 axis or by recruiting Treg cells into the tumor. Consistent with these results, the survival analyses revealed that the TCGA-HNSC cohort patients with higher LAMP3^+^ DC infiltration levels had worse OS (log-rank test, *P* = 0.05) (Fig. 7i).

Performing flow cytometry on seven additional samples from patients with HPSCC verified that LAMP3^+^ DCs were present in the tumor and expressed higher PD-L1, CD83, and CCL19 levels than LAMP3^–^ DCs. Additionally, tumor tissues had a higher proportion of LAMP3^+^ DCs than adjacent tissues (*P* < 0.05, Student’s t-test, Fig. 7j–k). Furthermore, LAMP3^+^ DC infiltration level in tumor tissues was positively correlated with Treg cells (Fig. 7l; *R* = 0.64, *P* = 0.03). Importantly, we found a strong positive correlation between PD-L1^+^ LAMP3^+^ DCs and CD8^+^ PD-1^+^ T cells (Fig. 7l; *R* = 0.66, *P* = 0.03), further confirming that LAMP3^+^ DCs may bind to PD-1 of CD8 T cells through their surface PD-L1 and promote CD8 T cell exhaustion in the TME, consistent with our scRNA-seq data. Finally, SCENIC analysis revealed that ETV3, HIVEP1, RELB, FOXO1, NF-κB 2, and ETS1 activities were upregulated in LMAP3^+^ DCs (Fig. S6c), being related to elevated immunosuppressive molecule expression and DC maturation [55].

## Discussion

Intratumoral heterogeneity is a major oncologic challenge. Here, we identified and validated six major mEpC subsets in HPSCC at single-cell resolution and revealed distinct cellular states and biological behaviors among the mEpC subsets via comprehensive bioinformatics analysis. We found that the different mEpC subsets in HPSCC have unique spatial localizations; the p-EMT mEpCs (metastatic cancer-2 cells) are localized at the leading margin of the primary tumor and close to the CAFs in the surrounding TME, which may induce collective migration of cell populations and promote local tumor invasion and lymph node metastasis [56]. Puram et al. identified tumor cells undergoing p-EMT in oral cancer using scRNA-seq and identified p-EMT as an independent adverse clinical feature predictor in oral squamous cell carcinoma [9]. Here, we identified p-EMT mEpCs in HPSCC for the first time, revealed their invasion and metastasis biology, and showed that p-EMT mEpCs are closely related to a poor prognosis. This study highlights the prevalence and importance of p-EMT-programmed cell subsets in HPSCC, revealing that HPSCC mEpCs have significant intratumor heterogeneity, and different types of mEpCs have different histological features and biological behaviors, which may affect treatment selection and prognosis. Precise treatment of mEpC subgroups may be the key for improving the prognosis of patients with HPSCC.

We found that mCAFs, marked by FAP, are the predominant fibroblast subset in HPSCC and closely associated with patient prognosis. Our analyses revealed extensive crosstalk between mCAFs and mEpCs via multiple cancer-related pathways, including Notch, Wnt, and TGF-β. These stemness signaling pathways are known to cause TME remodeling and modulate tumor progression [57]. In particular, p-EMT metastatic cancer-2 cells located at the tumor front exhibited stronger signaling crosstalk with mCAFs than with other mEpCs, mediated by chemokines and growth factors, such as the TIMP1–FGFR2 and HGF–CD44 pairs, and interactions such as those between the EMT-promoting TGF-β –TGF-β receptor, FGF2–FGFR3, and CXCL12–CXCR4 pairs. Furthermore, mCAFs were enriched in angiogenesis-related pathways that send multiple angiogenesis-related signals to blood ECs, including PGF, VEGFA, and PDGF, indicating that mCAFs are the key CAFs in promoting tumor angiogenesis and inducing tumor growth and metastasis in HPSCC in vivo [58]. Overall, these data suggest that mCAFs are involved in multiple steps during the metastatic process of HPSCC tumor cells, reflecting the important potential role of mCAFs in promoting malignant progression and invasive metastasis in HPSCC. The precise tumor promotion mechanism of mCAFs requires further studies and may provide new research directions for clinical translation and investigating tumor metastasis.

Remarkably, CAFs exhibit significant intertumor heterogeneity between different tumor types. Unlike in HPSCC, in intrahepatic cholangiocarcinoma, vascular CAFs, but not mCAFs, are key subsets for intrahepatic cholangiocarcinoma progression [59]. Conversely, inflammatory CAFs, which secrete CXCL12 and IL-6 in bladder carcinoma, are essential for inducing a bladder carcinoma tumor-immunosuppressive microenvironment and promoting bladder carcinoma progression [60]. A tumor-specific CST1^+^ myofibroblast subset with prognostic value and potential biological significance exists in esophageal squamous cell carcinoma [61]. Tumor-specific subsets of key CAFs exist in different solid tumors, and their key signaling pathways vary depending on the tumor type and tissue. Therefore, understanding this diversity among different tumors is crucial for understanding the tumor nature and developing specific therapeutic approaches.

Costa et al. [62] identified four CAF subsets in breast cancer using FACS, among which CAF-S1 is significantly associated with macrophage infiltration and promotes immunosuppressive microenvironment formation in breast cancer and was the only FAP-positive subset. This is similar to results obtained for mCAFs in HPSCC. Here, mCAFs strongly correlated and interacted with SPP1^+^ TAMs. Similar to SPP1^+^ macrophages in colon cancer [46], SPP1^+^ TAMs in HPSCC display pro-angiogenic, pro-tumorigenic, and pro-metastatic properties. Notably, cell communication analysis revealed that SPP1^+^ TAMs specifically interact with mCAFs through SPP1–CD44 and CD74–APP molecular interactions, which are critical for shaping the immunosuppressive and metastatic TME [52]. Furthermore, SPP1^+^ TAMs promote mCAF activation by secreting IL-1β or TGF-β1, which facilitates co-mediation of ECM remodeling by SPP1^+^ TAMs and mCAFs to form a pro-tumor fiber microenvironment that impedes lymphocyte infiltration. These results imply that mCAFs in HPSCC may be synergistically involved in TME remodeling with SPP1^+^ TAMs and highlight the importance of their extensive crosstalk for creating an immunosuppressive TME that promotes tumor angiogenesis and progression. Future studies should further elucidate how mCAFs and SPP1^+^ TAMs participate in tumor metastasis/progression and identify potential therapeutic targets.

Another noteworthy myeloid cell subset is LAMP3^+^ DCs, which have rarely been described in HNSCC scRNA-seq studies. As reported by Zhang et al. in hepatocellular carcinoma [63], LAMP3^+^ DCs in HPSCC contribute to the formation of a immunosuppressive TME and assist tumors in evading immune surveillance by T cells. Specifically, LAMP3^+^ DCs can recruit Tregs into the TME by expressing CCL17/CCL19 and inhibiting CD8^+^ T cell function via the CD274/PDCD1LG2–PDCD1 axis. We further confirmed the strong correlation between PD-1-expressing T cells and PD-L1-expressing LAMP3^+^ DCs in HPSCC tumor tissues using FACS, providing further evidence for their interactions. The immunomodulatory role of LAMP3^+^ DCs in lymphocytes in HPSCC suggests a potential immunotherapy target.

Unlike previous HNSCC scRNA-seq studies that focused on intertumor heterogeneity of malignant or immune cells [8–11], we mainly focused on elucidating the diversity and functions of different CAF subsets within HPSCC. We revealed the distinct biological properties and spatial heterogeneity of mEpC subsets and the complex tumor cellular ecosystem, highlighting the active and extensive crosstalk between mCAFs and mEpCs, stromal cells, and immune cells, suggesting that the cell communication network centered on mCAFs may participate in promoting malignant tumor progression and forming an immunosuppressive TME. We also identified a LAMP3^+^ DC subset with immunosuppressive effects in HPSCC. However, the small sample size is a notable limitation, and our findings must be validated in a larger cohort before the findings can reliably guide any development of therapy.

In conclusion, we provide a comprehensive transcriptomic picture of human HPSCC at single-cell resolution and a valuable resource for elucidating HNSCC diversity. We believe that this study will provide new directions for researching the mechanisms underlying HPSCC TME promotion in cancer progression and contribute to the search for novel molecular therapeutic targets.

### Supplementary Information

**Additional file 1: Table S1.** Clinical characteristics of six HPSCC patients in this study. **Table S2.** Basic information of single-cell RNA sequencing. **Table S3.** Signature genes used to define M1, M2, angiogenesis, and phagocytosis phenotypes, related to Fig. 6. **Table S4.** Signature genes used to define activation, migration, and tolerogenic phenotypes, related to Fig. 7.

**Additional file 2: Fig. S1.** Expression of marker genes for major cell types. Eleven major cell types were identified: epithelial cells (EPCAM+), endothelial cells (VWF+), fibroblasts (DCN+), T/NKT cells (CD3D+), B cells (MS4A1+), plasmocytes (JCHAIN), pDC cells (LILRA4), mast cells (TPSAB1+), myeloid cells (LYZ+), and neutrophils (FCGR3B+). **Fig. S2.** Copy number variations and characterization of malignant epithelial cells in HPSCC. a UMAP plot of epithelial cells (EpCs) colored by cluster (left) and patient (right). b Violin plots showing marker gene expression in EpCs subsets. c Copy number variations (CNVs) evaluated per cell using InferCNV. Immune and stromal cells were used as references. RNA velocities visualized with UMAP. EpCs (d) and fibroblasts (e) using Gaussian smoothing on a regular grid. f Scatter plots show expression of the top five highly expressed regulons in each malignant EpC subset, estimated using SCENIC. Kaplan–Meier curve of overall survival (OS) in the TCGA-HNSC cohort stratified by the optimal cut-off point for p-EMT marker gene (g), EMT marker gene (h), migratory proliferative marker gene (i), keratinocyte differentiation marker gene (j), and proliferative-related gene (k) expression levels. *P*-values were calculated using the two-sided log-rank test. **Fig. S3.** Heterogeneity and characterization of fibroblast in HPSCC. a UMAP plot of fibroblasts colored by cluster. b Bubble heatmap showing marker gene expression in pericytes. Dot size indicates the fraction of expressing cells, colored according to expression normalized by z-score. c Comparison of the absolute infiltration proportion of pericytes between paired normal (*n* = 43) and tumor tissues (*n* = 43) in the TCGA-HNSC cohort. Kaplan–Meier curve of the OS in the TCGA-HNSC cohort stratified by the optimal cut-off point for pericyte infiltration. *P*-values were calculated using the two-sided log-rank test. d Representative images showing multiplex immunohistochemistry (mIHC) staining of FAP in HPSCC tumor samples, in merged channels. Scale bar represents 50 μm. e Representative images showing mIHC staining of panCK and FAP in HPSCC tumor samples, in merged channels. Scale bar represents 20 μm. f Differences in the activities of hallmark pathways between different fibroblast subsets, scored using GSVA. g Representative plots of tumor-derived fibroblast (cancer-associated fibroblast, CAF) and normal tissue-derived fibroblast (normal fibroblast, NF). h Heatmap of *t*-values for the AUC scores of expression regulation based on transcription factor expression of fibroblast subsets, estimated using SCENIC. i UMAP plots of TF expression (up) and AUC scores (down). **Fig. S4.** Cell–cell interactions in HPSCC of fibroblast subsets and malignant epithelial cell subsets. **Fig. S5.** Expression of marker genes for myeloid and characteristics of SPP1^+^/C1QC^+^ TAM pathway. a Expression of marker genes for myeloid lineage cells. b GSEA shows top enriched pathways in SPP1^+^ TAM and C1QC^+^ TAM. NES denotes the normalized enrichment score. **Fig. S6.** Distribution of dendritic cell subsets and the relationship between DC and T cell subsets. a Bar graph showing abundance of dendritic cell (DC) subsets that differ between tumors (red) and adjacent normal tissues (blue) from scRNA-seq data. b Correlation between LAMP3^+^ DC signature and Treg, T_ex_ and effector T cell signatures in the TCGA-HNSC cohort. Coefficients were calculated with Pearson’s correlation analysis. c Heatmap of *t*-values for the AUC scores of expression regulation based on transcription factor expression of DC subsets, estimated using SCENIC.

**Additional file 3: Fig. S7.** The full-lengthe gel images of N-cadherin, E-cadherin, Vimentin, GADPH and Twist.

## Data Availability

The data generated in this study are available upon request from the corresponding author. Data from the TCGA cohort are openly available in the TCGA repository at https://portal.gdc.cancer.gov/.

## Abbreviations

HPSCC: Hypopharyngeal squamous cell carcinoma
TME: Tumor microenvironment
CAF: Cancer-associated fibroblasts
mCAF: Extracellular matrix cancer-associated fibroblast
TAM: Tumor-associated macrophages
EpC: Epithelial cell
mEpC: Malignant epithelial cell
DEG: Differentially expressed gene
EMT: Epithelial-mesenchymal transition
OS: Overall survival
scRNA-seq: Single-cell RNA sequencing
GEMs: Gel-beads in emulsions
CNVs: Copy number variants
TF: Transcription factor
GSVA: Gene set variation analysis
TCGA: The Cancer Genome Atlas
mIHC: Multiplex immunohistochemistry
HNSCC: Head and neck squamous cell carcinoma
CM: Cultured media
EC: Endothelial cell
FAP: Fibroblast activation protein
ECM: Extracellular matrix
EndMT: Endothelial-mesenchymal transition
