# Supplementary material for: Single-cell RNA sequencing reveals pro-invasive cancer-associated fibroblasts in hypopharyngeal squamous cell carcinoma
Source: Cell Commun Signal. 2023 Oct 18;21:292. doi: 10.1186/s12964-023-01312-z (PMC10585865; doi:10.1186/s12964-023-01312-z)
Supplement: Supplementary file 2 — Additional file 1: Table S1. Clinical characteristics of six HPSCC patients in this study. Table S2. Basic information of single-cell RNA sequencing. Table S3. Signature genes used to define M1, M2, angiogenesis, and phagocytosis phenotypes, related to Fig. 6. Table S4. Signature genes used to define activation, migration, and tolerogenic phenotypes, related to Fig. 7. [file 12964_2023_1312_MOESM1_ESM.docx]

**Table S1. Clinical characteristics of 6 HPSCC patients in this study.**

| **Patient** | **Sample** | **Age/Sex** | **Primary site** | **TNM Stage^*^** | **Tumor grade** |
| --- | --- | --- | --- | --- | --- |
| HPSCC 1 | P1-T | 68/M | Right pyriform sinus | T4aN2cM0 | Poorly-moderately differentiated, Grade Ⅲ |
| HPSCC 2 | P2-T | 60/M | Left pyriform sinus | T4aN0M0 | moderately differentiated, Grade Ⅱ |
| HPSCC 3 | P3-T | 60/M | Bilateral pyriform sinus | T4aN2cM1 | Poorly-moderately differentiated, Grade Ⅲ |
| HPSCC 4 | P4-T | 57/M | Posterior wall of hypopharynx | T2N2bM0 | Well- moderately differentiated, Grade Ⅱ |
|  | P4-N |  |  |  |  |
| HPSCC 5 | P5-T | 70/M | Right pyriform sinus | T3N0M0 | Poorly differentiated, Grade Ⅲ |
|  | P5-N |  |  |  |  |
| HPSCC 6 | P6-T | 63/M | Right pyriform sinus | T4bN3aM0 | Poorly differentiated, Grade Ⅲ |

^*^: Pathological diagnosis and TNM stage of HPSCC were determined according to the 8th edition of the International Union against Cancer (UICC) and American Joint Committee on Cancer (AJCC) staging system.

**Table S2. Basic information of single cell RNA sequencing.**

| **Sample** | **Estimated Number of Cells** | **Mean Reads per Cell** | **Median Genes per Cell** | **Total Genes Detected** | **Saturation** |
| --- | --- | --- | --- | --- | --- |
| P1-T | 10088 | 32204 | 1403 | 29336 | 54.9% |
| P2-T | 11783 | 41008 | 1640 | 25800 | 59.9% |
| P3-T | 11365 | 43759 | 1257 | 25326 | 57% |
| P4-T | 7395 | 43941 | 1057 | 32751 | 54.8% |
| P4-N | 12862 | 24769 | 2050 | 33988 | 27.8% |
| P5-T | 8923 | 59246 | 1166 | 24435 | 74.8% |
| P5-N | 11234 | 46219 | 2156 | 25924 | 62.5% |
| P6-T | 11961 | 25786 | 2402 | 31646 | 24.7% |

**Table S3. Signature genes used to define M1, M2, angiogenesis and phagocytosis phenotypes, related to Figure 6**

| M1 Polarization | M2 Polarization | Angiogenesis | Phagocytosis |
| --- | --- | --- | --- |
| IL23 | IL4R | CCND2 | MRC1 |
| TNF | CCL4 | CCNE1 | CD163 |
| CXCL9 | CCL13 | CD44 | MERTK |
| CXCL10 | CCL20 | CXCR4 | C1QB |
| CXCL11 | CCL17 | E2F3 |  |
| CD86 | CCL18 | EDN1 |  |
| IL1A | CCL22 | EZH2 |  |
| IL1B | CCL24 | FGF18 |  |
| IL6 | LYVE1 | FGFR1 |  |
| CCL5 | VEGFA | FYN |  |
| IRF5 | VEGFB | HEY1 |  |
| IRF1 | VEGFC | ITGAV |  |
| CD40 | VEGFD | JAG1 |  |
| IDO1 | EGF | JAG2 |  |
| KYNU | CTSA | MMP9 |  |
| CCR7 | CTSB | NOTCH1 |  |
|  | CTSC | PDGFA |  |
|  | CTSD | PTK2 |  |
|  | TGFB1 | SPP1 |  |
|  | TGFB2 | STC1 |  |
|  | TGFB3 | TNFAIP6 |  |
|  | MMP14 | TYMP |  |
|  | MMP19 | VAV2 |  |
|  | MMP9 | VCAN |  |
|  | CLEC7A | VEGFA |  |
|  | WNT7B |  |  |
|  | FASL |  |  |
|  | TNFSF12 |  |  |
|  | TNFSF8 |  |  |
|  | CD276 |  |  |
|  | VTCN1 |  |  |
|  | MSR1 |  |  |
|  | FN1 |  |  |
|  | IRF4 |  |  |

**Table S4. Signature genes used to define activation, migration and tolerogenic phenotypes, related to Figure 7**

| DC activation | DC migration | DC tolorgenic |
| --- | --- | --- |
| FSCN1 | GAL3ST | STAB1 |
| BIRC3 | NUDT17 | ANXA1 |
| LAMP3 | ITGB8 | IDO1 |
| CCL19 | ADCY6 | IL10 |
| LAD1 | ENO2 | IL27 |
| MARCKS | IL15RA | STAT3 |
| TNFAIP2 | SOCS2 | ENTPD1 |
| CCR7 | IL15 | SOCS2 |
| CCL22 | STAP2 | CCR7 |
| MARCKSL1 | PHF24 | CD274 |
| EBI3 | ANKRD33B | FTL |
| TNFRSF11B | INSM1 | IRF1 |
| NUB1 | ANXA3 | IRF2 |
| INSM1 | ARHGAP28 |  |
| RAB9A | RNF115 |  |
| LY75 | ADORA2A |  |
| SIAH2 | EXTL1 |  |
| POGLUT1 | SPSB |  |
| KDM2B | SLC22A23 |  |
| MGLL | RABGAP1 |  |
| TXN | GYG1 |  |
| MLLT6 | DAP |  |
| KIF2A | OGFR |  |
| GRSF1 | GYG2 |  |
| FAM49A | CCSER2 |  |
| PLEKHG1 | TMEM123 |  |
| SOCS2 | NET1 |  |
| RFTN1 | GPR52 |  |
| AC009812.4 | SLCO5A1 |  |
| BMP2K | FAH |  |
| NAV1 | CLU |  |
| IL7R | PCGF5 |  |
| ID2 | SAMSN1 |  |
| CCL17 | CDKN2B |  |
| PPP1R9B | BMP2K |  |
| NRP2 | ZC2HC1A |  |
| TUBB6 | SERINC5 |  |
| ARNTL2 | HIVEP1 |  |
| UVRAG | CNR1 |  |
| TXNDC11 | CNR2 |  |
| MREG |  |  |
| BTG1 |  |  |
