# Supplementary figures and images for: Single-cell RNA sequencing reveals pro-invasive cancer-associated fibroblasts in hypopharyngeal squamous cell carcinoma
Source: Cell Commun Signal. 2023 Oct 18;21:292. doi: 10.1186/s12964-023-01312-z (PMC10585865; doi:10.1186/s12964-023-01312-z)

Expression of marker genes

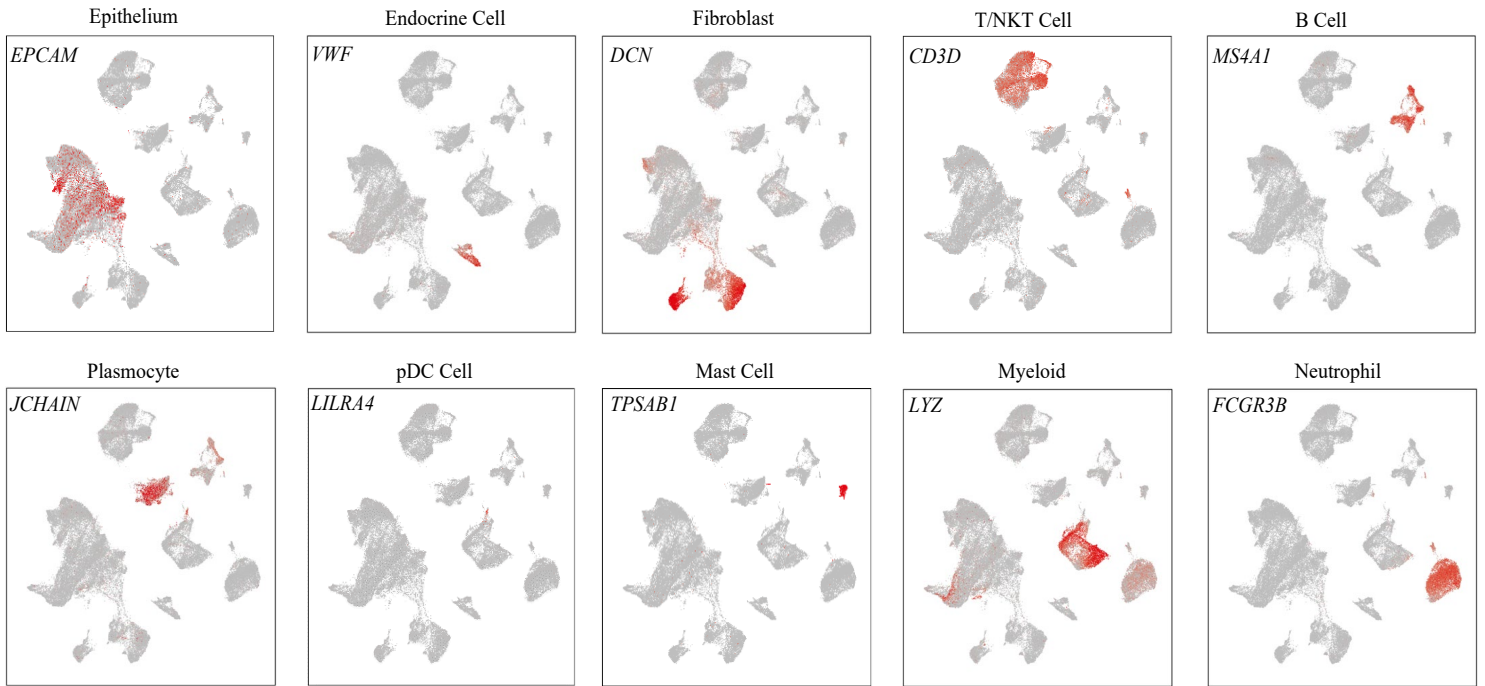



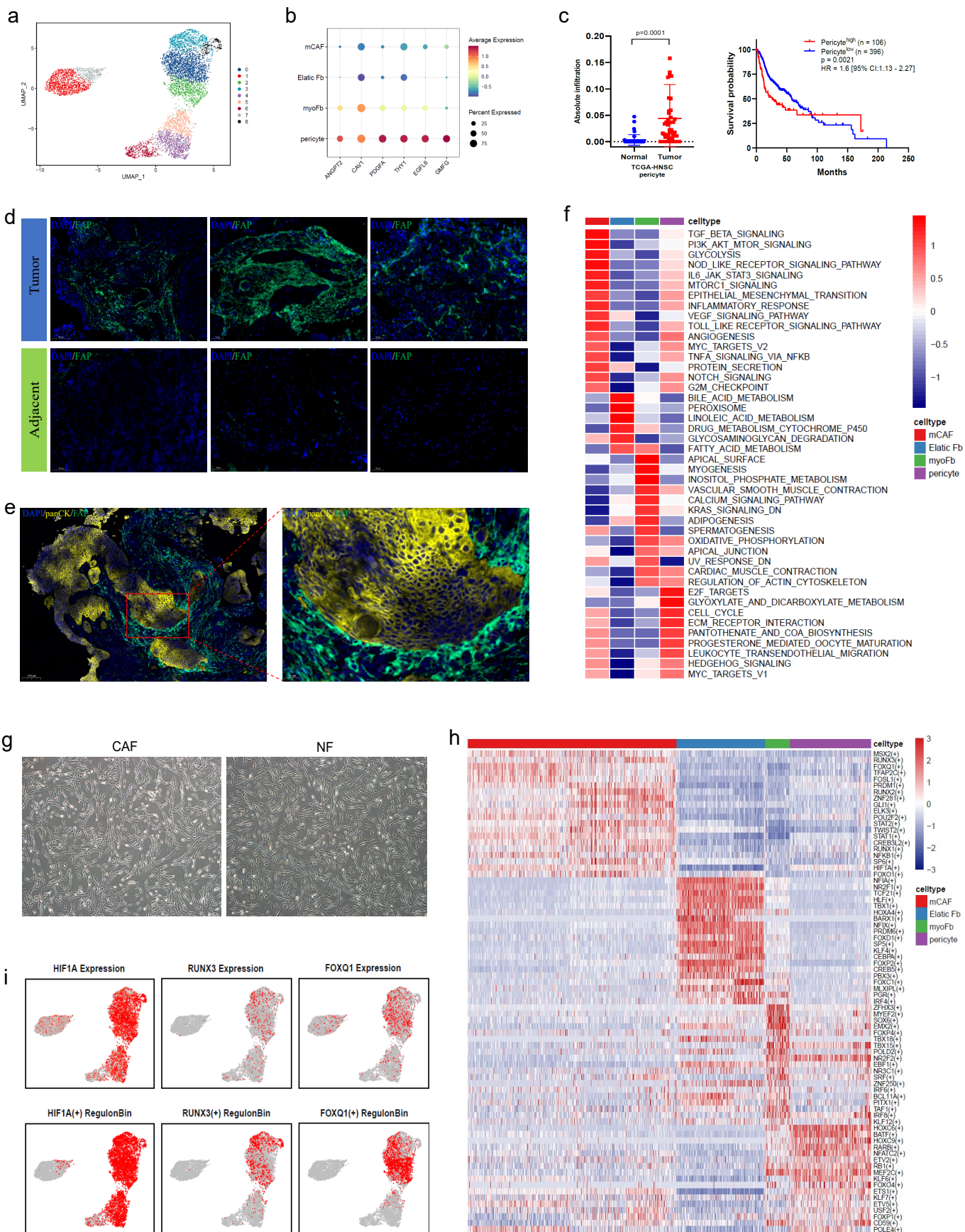

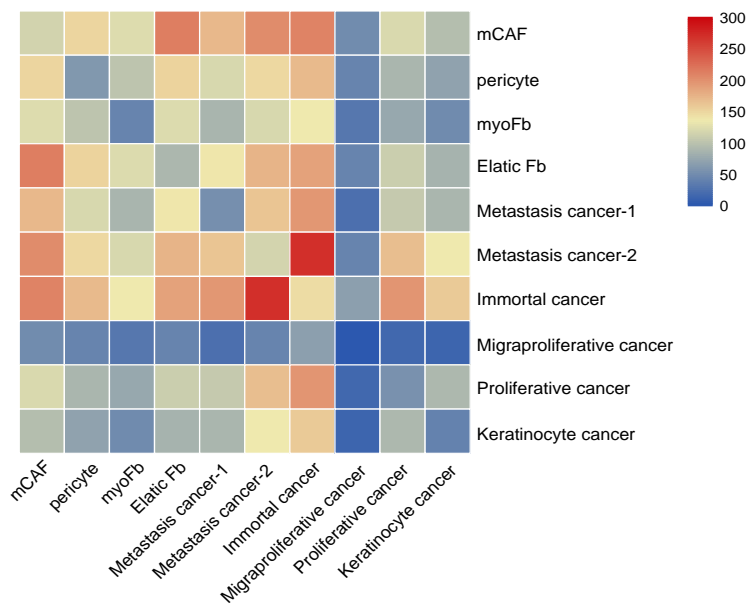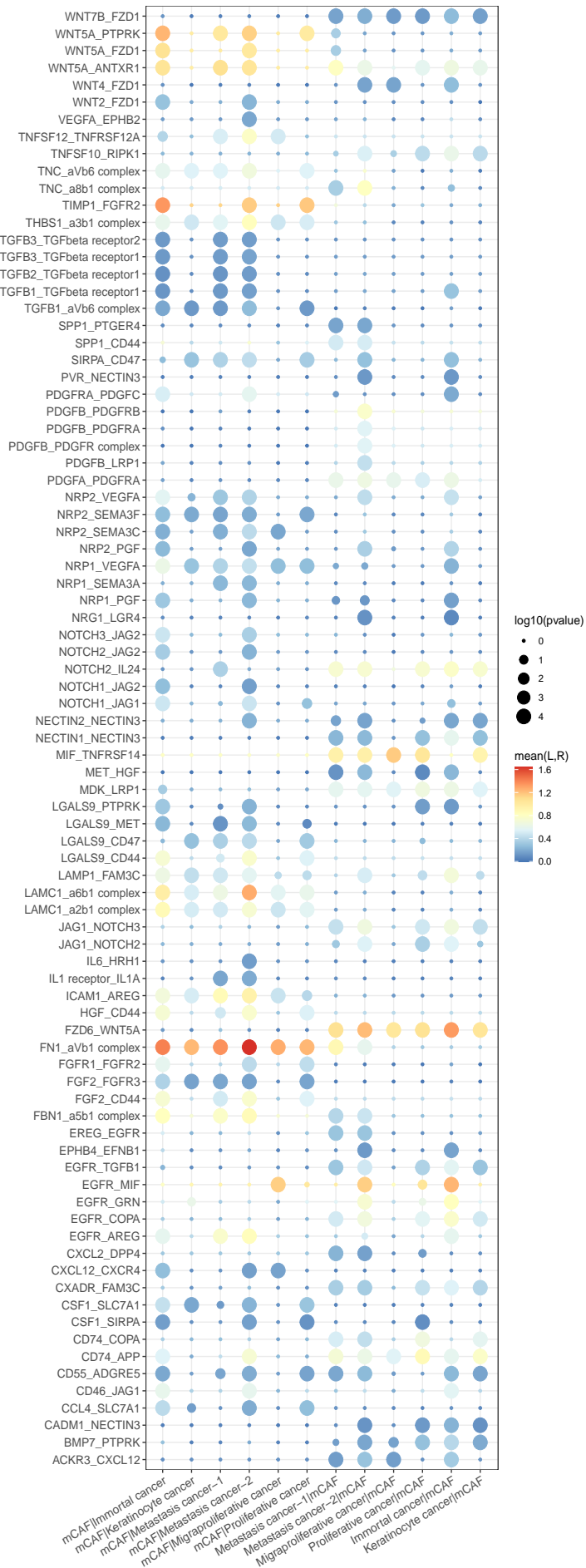

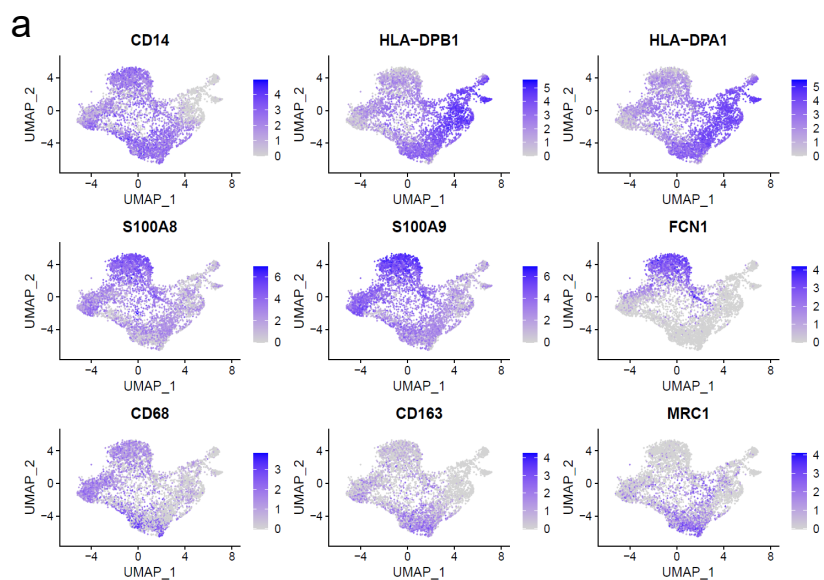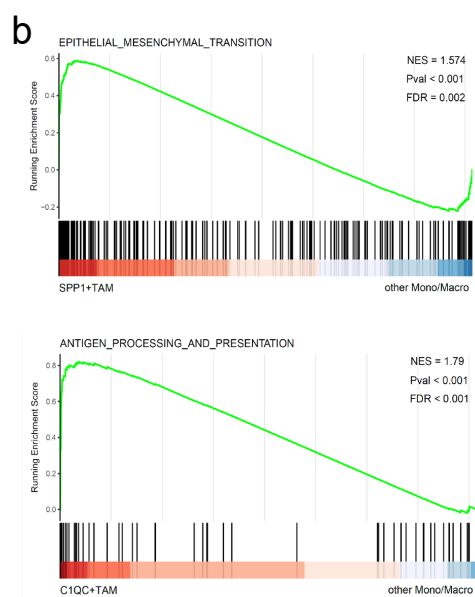

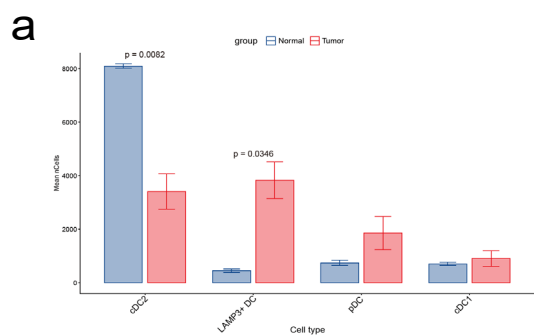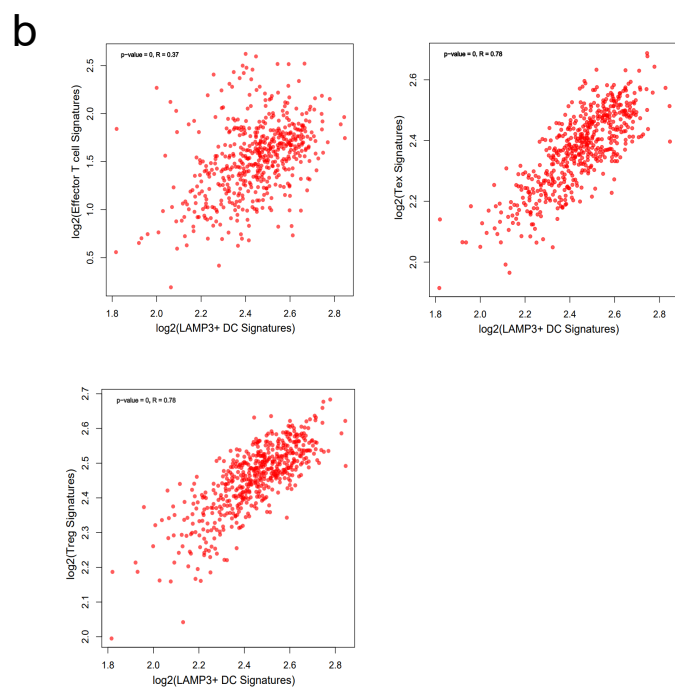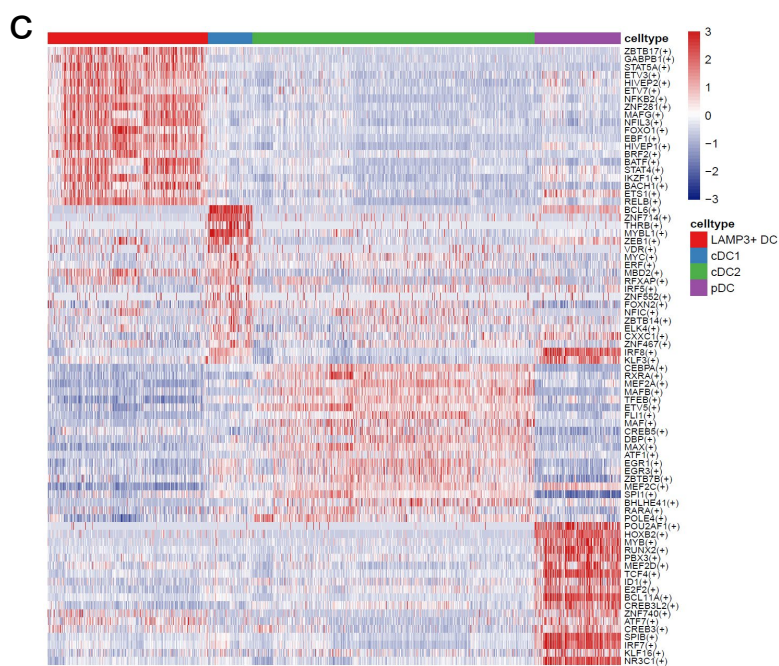

Supplement: Supplementary file 3 — Additional file 2: Fig. S1. Expression of marker genes for major cell types. Eleven major cell types were identified: epithelial cells (EPCAM+), endothelial cells (VWF+), fibroblasts (DCN+), T/NKT cells (CD3D+), B cells (MS4A1+), plasmocytes (JCHAIN), pDC cells (LILRA4), mast cells (TPSAB1+), myeloid cells (LYZ+), and neutrophils (FCGR3B+). Fig. S2. Copy number variations and characterization of malignant epithelial cells in HPSCC. a UMAP plot of epithelial cells (EpCs) colored by cluster (left) and patient (right). b Violin plots showing marker gene expression in EpCs subsets. c Copy number variations (CNVs) evaluated per cell using InferCNV. Immune and stromal cells were used as references. RNA velocities visualized with UMAP. EpCs (d) and fibroblasts (e) using Gaussian smoothing on a regular grid. f Scatter plots show expression of the top five highly expressed regulons in each malignant EpC subset, estimated using SCENIC. Kaplan–Meier curve of overall survival (OS) in the TCGA-HNSC cohort stratified by the optimal cut-off point for p-EMT marker gene (g), EMT marker gene (h), migratory proliferative marker gene (i), keratinocyte differentiation marker gene (j), and proliferative-related gene (k) expression levels. P-values were calculated using the two-sided log-rank test. Fig. S3. Heterogeneity and characterization of fibroblast in HPSCC. a UMAP plot of fibroblasts colored by cluster. b Bubble heatmap showing marker gene expression in pericytes. Dot size indicates the fraction of expressing cells, colored according to expression normalized by z-score. c Comparison of the absolute infiltration proportion of pericytes between paired normal (n = 43) and tumor tissues (n = 43) in the TCGA-HNSC cohort. Kaplan–Meier curve of the OS in the TCGA-HNSC cohort stratified by the optimal cut-off point for pericyte infiltration. P-values were calculated using the two-sided log-rank test. d Representative images showing multiplex immunohistochemistry (mIHC) staining of FAP in [file 12964_2023_1312_MOESM2_ESM.pdf]
