# Supplementary material for: Single-cell RNA sequencing reveals pro-invasive cancer-associated fibroblasts in hypopharyngeal squamous cell carcinoma
Source: Cell Commun Signal. 2023 Oct 18;21:292. doi: 10.1186/s12964-023-01312-z (PMC10585865; doi:10.1186/s12964-023-01312-z)
Supplement: Supplementary file 4 — Additional file 3: Fig. S7. The full-lengthe gel images of N-cadherin, E-cadherin, Vimentin, GADPH and Twist. [file 12964_2023_1312_MOESM3_ESM.docx]

**SNU1076 cell lines**

**Fadu cell lines**

**Medium**

**Medium**

**NF^CM^**

**CAF^CM^**

**NF^CM^**

**CAF^CM^**


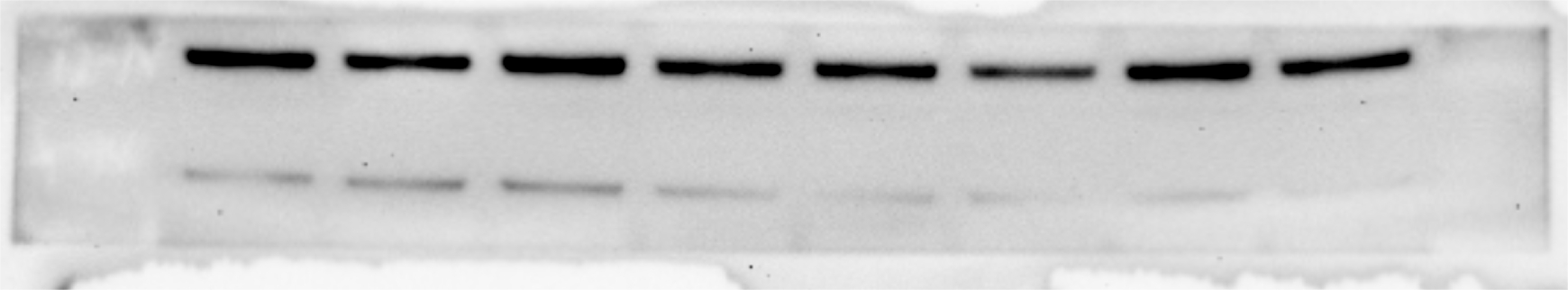

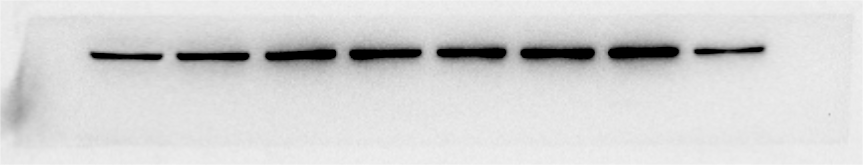

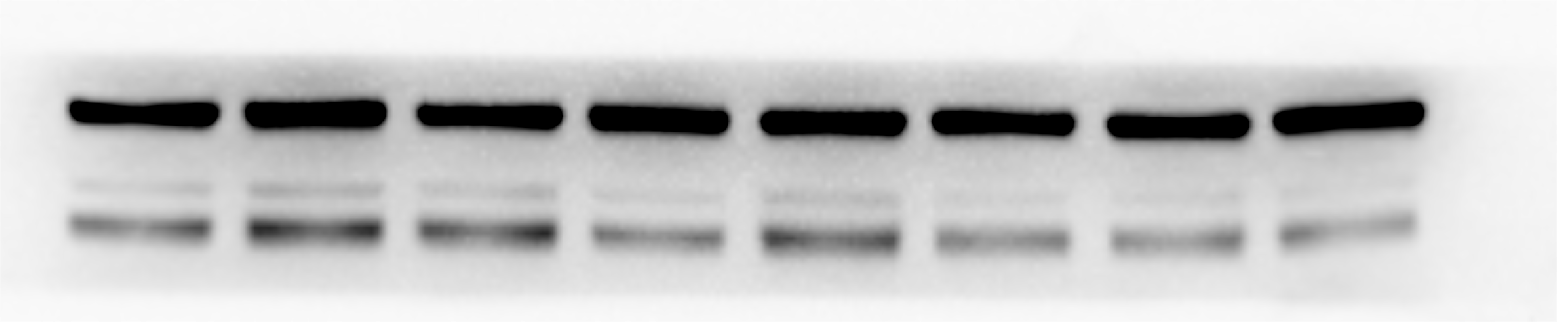

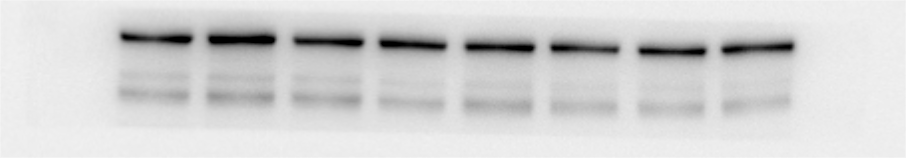


**E-cadherin**

**N-cadherin**


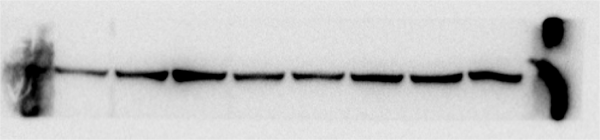

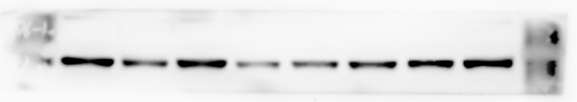


**Vimentin**


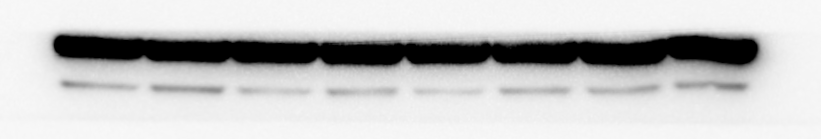

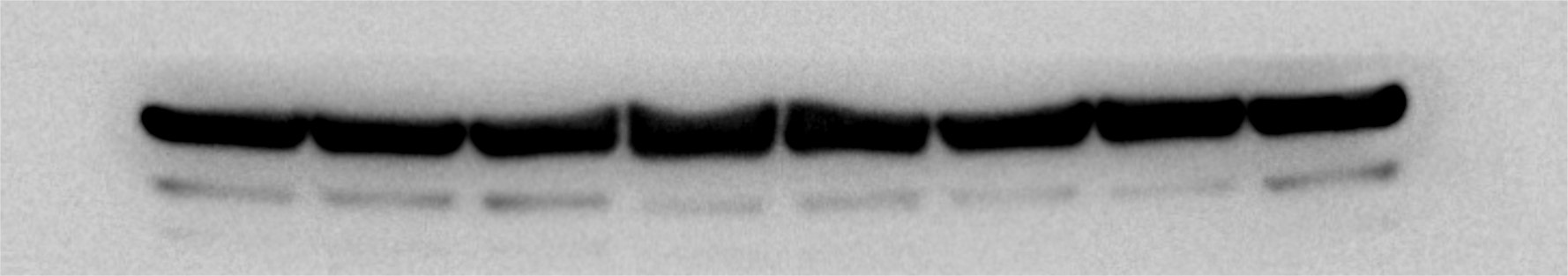


**GADPH**


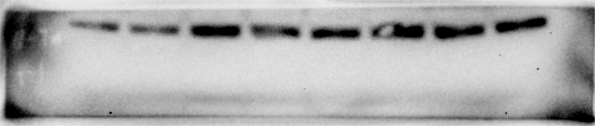

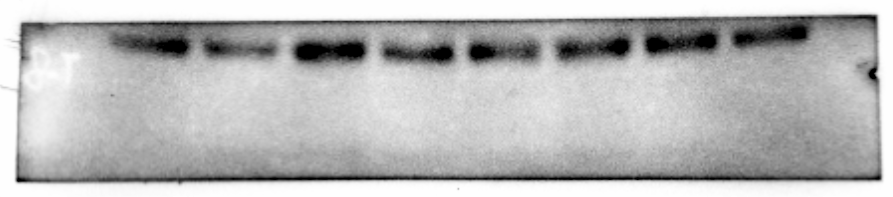


**Twist**
